# An innovative medical waste management system in a smart city using XAI and vehicle routing optimization

**DOI:** 10.12688/f1000research.138867.1

**Published:** 2023-08-31

**Authors:** Zineb Boudanga, Siham benhadou, Hicham Medromi

**Affiliations:** 1 Engineering research laboratory (LRI), System Architecture Team (EAS), National and high school of electricity and mechanic (ENSEM), University Hassan II Casablanca, Casablanca, Grand Casablanca, Morocco; 2 Fondation de Recherche de Developpement et d'Innovation en Sciences et Ingenierie, Casablanca, Grand Casablanca, Morocco

**Keywords:** Medical waste management, Smart city, IoT, Explainable AI (XAI), GA, TDVRPTW

## Abstract

**Background:** The management of medical waste is a complex task that necessitates effective strategies to mitigate health risks, comply with regulations, and minimize environmental impact. In this study, a novel approach based on collaboration and technological advancements is proposed.

**Methods:** By utilizing colored bags with identification tags, smart containers with sensors, object recognition sensors, air and soil control sensors, vehicles with Global Positioning System (GPS) and temperature humidity sensors, and outsourced waste treatment, the system optimizes waste sorting, storage, and treatment operations. Additionally, the incorporation of explainable artificial intelligence (XAI) technology, leveraging scikit-learn, xgboost, catboost, lightgbm, and skorch, provides real-time insights and data analytics, facilitating informed decision-making and process optimization.

**Results:** The integration of these cutting-edge technologies forms the foundation of an efficient and intelligent medical waste management system. Furthermore, the article highlights the use of genetic algorithms (GA) to solve vehicle routing models, optimizing waste collection routes and minimizing transportation time to treatment centers.

**Conclusions:** Overall, the combination of advanced technologies, optimization algorithms, and XAI contributes to improved waste management practices, ultimately benefiting both public health and the environment.

## Introduction

Medical waste management (MWM) is a critical aspect of healthcare facilities that necessitates effective strategies to mitigate health risks, comply with regulations, and minimize environmental impact. Improper management and inadequate disposal of medical waste can lead to harmful outcomes for public health and the environment. ^1^ This underscores the urgent requirement for innovative methods aimed at improving the efficiency, safety, and sustainability of MWM systems.

Various scientific articles have emphasized the importance of advanced technologies in MWM. For example, a study conducted by Ref. 2 explored the application of radio-frequency identification (RFID) technology in MWM and highlighted its positive influence on waste disposal. The research revealed that integrating RFID technology with video monitoring and cloud storage can significantly mitigate the risk of medical waste loss or unauthorized recycling. Similarly, the study ^3^ investigated waste generation and management practices in the healthcare sector in Colombo, Sri Lanka, with the aim of reducing pollution. It emphasized the significance of positive attitudes, awareness, capabilities, and technology in improving waste management processes, encouraging healthcare organizations to invest in this area. Additionally, another study ^4^ highlighted the importance of blockchain technology in MWM and identified various clusters such as waste generation, storage, collection, treatment, and disposal. Building upon these technological advancements, a different study introduced a waste management innovation model called the “Four Joins of Power”, which emphasized community engagement, knowledge-sharing, collaboration, and network expansion as key pillars in effective waste management. ^5^ By employing a four-phase approach, including situation analysis, innovation development, trial, and assessment, the implementation of the “Four Joins of Power” innovation resulted in increased community knowledge and positive changes in waste management behavior among participants. These articles provide valuable insights into the potential of advanced technologies to improve the effectiveness and efficiency of MWM processes.

In this context, our article proposes a novel approach that integrates collaboration and technological advancements to optimize waste sorting, storage, and treatment operations. By harnessing advanced technologies such as sensor-based systems, Global Positioning System (GPS)-enabled vehicles, and explainable artificial intelligence (XAI) technology, this work aims to revolutionize the field of MWM. The study showcases the efficiency, safety, and environmental compliance achieved through the implementation of this smart MWM system. Furthermore, the article briefly mentions the use of vehicle routing models to optimize waste collection routes and minimize transportation time to treatment centers.

The article is structured as follows: the related work section provides relevant scientific literature on MWM and supporting evidence for the use of advanced technologies in waste management. The methods section presents a concise description of how the study was conducted. The proposed solution section details the algorithms and technologies employed, presents the findings of the study, and is followed by a discussion that analyzes and interprets the results. Finally, the conclusion section summarizes the key findings and highlights the significance of the study’s contributions to the field of MWM.

## Related work

This study is based on a comprehensive literature search, focusing on three specific areas, namely (a) MWM System (MWS), (b) Intelligent MWS, and (c) XAI applied to MWM. An extensive literature review is presented in this section, outlining the existing gaps and the motivation behind this study.

### MWM process

Medical waste, which includes materials that are infectious or contain toxic chemicals, can be harmful to people and the environment. Hospitals and health centers generate a significant amount of this waste each year, and it is essential to manage it properly to ensure the safety of patients, healthcare workers, and the community. ^6^

Improper MWM can pose serious health and environmental risks, including contamination, pollution, and exposure to hazardous materials. It is therefore crucial to handle, treat, and dispose of medical waste safely to protect public health and the environment.

MWM encompasses various practical components, including waste collection, waste separation, waste recovery, and waste scheduling. Furthermore, the development of efficient strategies and methodologies is crucial in establishing an effective framework for waste management within MWM. ^7^ These aspects have been extensively explored and analyzed in the relevant literature.

In the referenced study, ^8^ the focus is on investigating the sustainability challenges associated with MWM in developing countries across Africa. The authors specifically analyze various aspects, including the proper handling of waste within hospitals and health facilities, as well as the transportation and storage of medical waste. In addition, they examined the impact of underfunded health systems, inadequate training, and lack of awareness of MWM policies and legislation. They proposed a management plan considering policy and fiscal aspects, collaboration between different institutions, the use of cost-effective and sustainable treatment methods, the establishment of an efficient supply chain and adequate storage.

In a related vein, researchers in Ref. 9 conducted a comprehensive meta-analysis that examined medical and healthcare waste management practices across 78 countries. Their findings indicated a noteworthy association between the rate of medical waste generation and factors such as the human development index, life expectancy, and health expenditure. Conversely, they discovered a negative correlation between medical waste generation and the environmental performance index. Furthermore, the study underscores the significance of promoting awareness among workers regarding best practices in waste management.

Although some authors use the latest technologies to address the risks of MWM, for example the authors of Ref. 10 proposed a decentralized blockchain-based system to automate medical waste processes and makes it transparent. Their solution consists of four components: medical equipment and supplies, waste centers, recycling plants and sorting factories.

Furthermore, the performance of MWM system has been recently interrupted and encountered a very serious situation due to the epidemic outbreak of the Coronavirus disease 2019 (COVID-19). ^11^ And the disposal of this new category of biomedical waste (COVID-19 waste) is a major global concern for public health and environmental sustainability, given the significant risk of pandemic spread. This article ^12^ reviews the technologies for disinfecting COVID-19 waste, from separate collection to the various physical and chemical treatment steps. The authors proposed chemical disinfection using a 1% NaOCl solution *in situ*, as well as microwave disinfection is to disinfect personal protection equipment (PPE) and wipes that can be recycled and reused, while incineration is useful to treat a larger volume of COVID-19 waste.

Moreover, to properly manage COVID-19 medical waste, the authors ^13^ designed a reverse logistics network to control the spread of the virus. In this regard, this study presented a tri-objective mathematical model to minimize the total cost, the risk associated with the network operations, and the maximum amount of uncollected waste. Also, this work ^14^ analyzed the existing MWM system in Korea and proposed measures to establish effective management of Covid-19 waste. The authors proposed the use of effective medical waste classification, reduction of medical waste generation and diversification of treatment methods as areas for improvement.

Within the cited literature, multiple studies have proposed various MWM systems. However, the absence of a standardized evaluation framework for assessing these systems remains evident. Addressing this gap, the research presented in Ref. 15 introduces an assessment framework for MWM based on guidelines established by the World Health Organization (WHO). The framework incorporates multi-criteria decision making (MCDM) techniques to model and evaluate the effectiveness of MWM practices. To demonstrate its applicability, the framework was implemented in eight private and public hospitals in Myanmar, enabling an assessment of their respective MWM practices. The results of this study show the urgent need for specific MWM laws and regulations, technology, expertise, and funding, as well as the need for risk awareness among health care staff. The authors also recommend the implementation of new environmentally friendly technologies and the encouragement of collaboration between public and private institutions. In addition, an analytical hierarchy process (AHP) methodology was used in this paper ^16^ to help each hospital unit to verify its environmental situation, as well as to specify the areas and processes that should be improved towards environmental sustainability.

To summarize, most of the literature cited above suggests sensitizing stakeholders to best practices in MWM and associated risks, collaborating among institutions to optimize resource utilization, and developing a comprehensive management framework from waste production to treatment. Our work will address these issues by proposing practical and environmentally friendly solutions.

### Smart waste management

Most cities aim to transform their infrastructure based on sustainability guidelines and practices. Specifically, smart technologies such as the Internet of Things (IoT) and blockchain are being used to maximize economic and social benefits and minimize environmental issues. ^17^^–^^19^ For instance, several research propose an IoT-based connected environment to better manage waste. ^20^

Firstly, the MWS which relies on sensors and other smart devices, is potentially more efficient in sorting waste. In this context, ^21^ exploit various types of sensors (proximity sensor, humidity sensor, gas sensor, and ultrasonic sensor, among others) to collect and sort waste. Indeed, they propose a waste segregator that can identify the type of waste and sort it into bins automatically. Also, the authors of Ref. 22 have proposed an IoT solution to sort medical waste. Their solution encompasses five-steps: waste image capture, data preprocessing, median filtering, contrast enhancement and segmentation. There is also an “iWASTE” solution based on cameras in waste bins cans for the detection and classification of medical waste using video recordings. ^23^ Moreover, ^24^ proposes a system encompassing real-time waste tracking sensors such as RFID, GPS, *etc.,* cloud computing for data storage and transmission, mobile application for monitoring and tracking. As well as a fuzzy method based categorization is performed to classify the waste according to specific criteria.

Secondly, there are solutions using robotics to optimize MWM. For example, ^25^ proposes a solution based on a robotic arm for waste sorting, ^26^ proposes a self-supporting vehicle with robotic hands used to collect waste.

In general, several models have been proposed for waste tracking and management, including smart bins, ^27^^,^^28^ a cloud-based data encryption and decryption method for a secure waste management system. ^29^ In Ref. 30 they propose a waste management platform with unique bin identifier and real-time tracking of waste levels, this platform is intended to facilitate waste tracking by multiple parties, such as government agencies and hospitals. Where Ref. 31 proposes an IoT infrastructure system incorporating more than data collection, data processing as well as management application integration for waste optimization.

The IoT is essential for MWM since it integrates the required technologies such as identification technologies, data acquisition, spatial technologies and communication technologies, in addition it must also integrate Artificial Intelligence (AI) methods allowing decision support. ^32^ However, in the related literature, the use of smart cities models in terms of medical waste is limited, with most work focusing on municipal waste management.

Our contribution goes beyond the use of IoT for waste sorting and tracking, as we have developed a comprehensive smart solution that covers the entire waste management process from generation to disposal. Our approach includes the deployment of smart devices in the hospital, external warehouse, transport vehicle, and waste processing unit, while leveraging AI and big data to optimize efficiency. Additionally, we have proposed a collaboration system among all stakeholders to ensure the success of the solution. Furthermore, to promote transparency and understanding among the involved parties, we have integrated XAI in our solution.

### XAI for medical waste

AI is becoming more and more prevalent in our daily lives, with intelligent systems being used for a variety of purposes such as recommending content and products, providing news, managing social media, delivering healthcare, and providing other public services. ^33^ However, the inner workings of these AI systems are not always transparent, and often do not provide enough information about how decisions are made. ^34^ Indeed, only the programmers of the AI algorithm understand how the system works. ^35^ Therefore, XAI is essential to allow end users to make effective decisions in different contexts, especially critical use cases to rely on the system. ^36^

Several works ^6^^,^^37^^–^^54^ have studied XAI in various domains and perspectives, *e.g.* healthcare, media and entertainment, education, transportation, finance, e-commerce, digital assistant, *etc.*

In healthcare, researchers are using XAI for disease diagnosis and health-related recommendation systems. ^37^^,^^38^ For media and entertainment, involves personalized recommendation systems, based on collected personal information. ^39^^–^^42^ Uses of XAI in education encompass smart tutoring systems, university admission decision making, and grade estimation systems. ^43^^–^^45^ The transportation domain includes navigation systems, applications for autonomous cars and flight planning for the aviation industry. ^46^^–^^48^ Financial applications of XAI research include the area of insurance, the possibility of financial fraud detection and loan application management. ^49^^,^^50^ For E-commerce, XAI is used as a useful marketing tool, or explained online purchase recommendations. ^51^^–^^53^ For digital assistants, there are applications of XAI-based interactive agents that are trustworthy and more user-centered. ^54^

In general XAI are widely used across different fields, but in MWM, there is no research in this direction, only our latest work ^6^ which is to propose an intelligent solution based on XAI so that stakeholders trust the choice of waste treatment, collection schedule, treatment methods, *etc.*

### Literature gap and contributions of this research

The literature review revealed that majority of the works for MWM do not integrate a comprehensive solution containing the different parties involved, each work deals with the problem from the point of view of either hospital, or treatment unit or waste collection and transportation. On the other hand, the research objective of management models for medical waste is to provide sound policy decisions and suggest operational strategies for designing the system in a cost-effective and environmentally sound manner. However, to the best of our knowledge, no research has been conducted to design a transparent, smart system for the management of medical waste and the resulting health risks. To fill the gaps in the literature, this work proposes an integrative model for effective management of medical waste.

The main objectives of this research are summarized as follows:

• Propose a model that integrates all the different parties involved in MWM, from waste generation to disposal.
• Incorporate smart technologies, AI, and big data into the proposed solution.
• Ensure transparency and explainability of the proposed solution by integrating XAI, so that all parties involved can understand the decision-making process.
• Optimize the vehicle routing problem for the collection and distribution of medical waste.

## Methods

**Figure 1.**
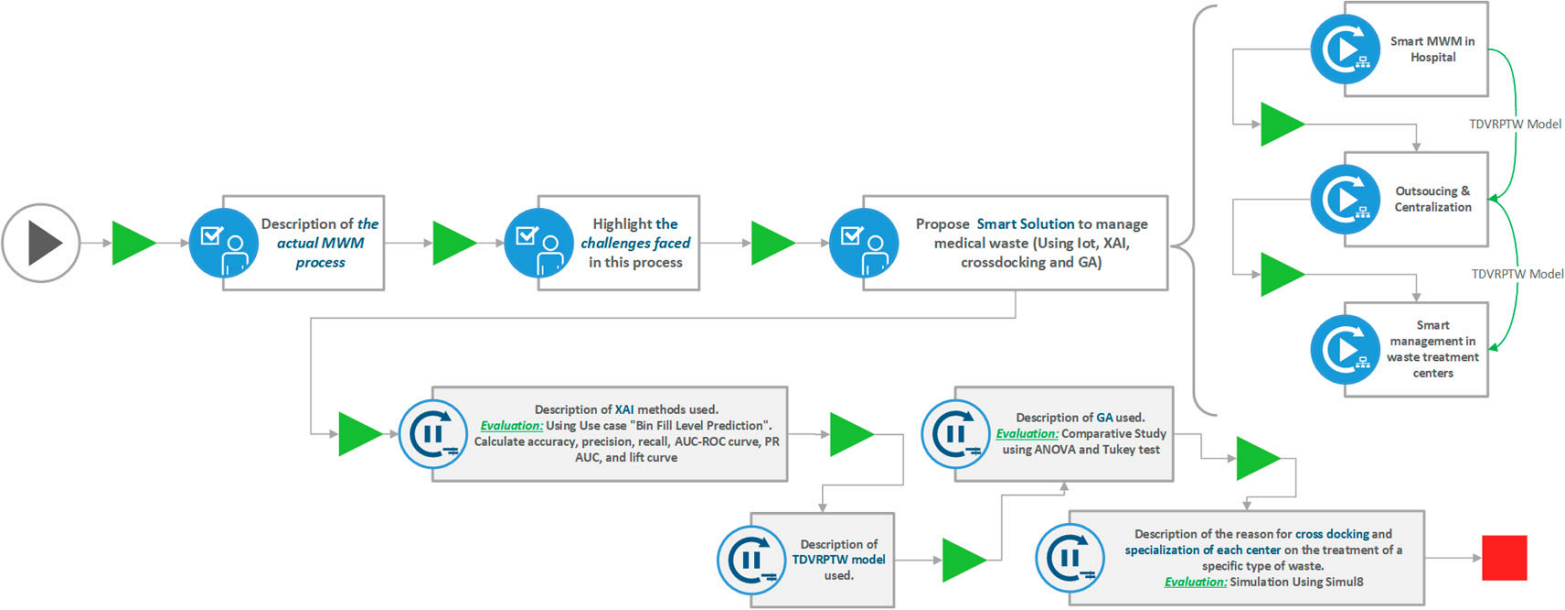
Overview of the methodology employed in this work.

The approach employed in our work aligns with the methodology outlined in this section (see Figure 1), which encompasses the process of addressing our research question, elucidating the implementation steps, substantiating the chosen experimental design, and detailing the analysis of obtained results.

### Current MWM process

We begin by providing a detailed overview of the actual MWM process in the context of Morocco as an example. This includes a step-by-step description of activities such as sorting and packaging, storage, transport, treatment, and disposal. We also highlight the key challenges associated with each stage, such as health risks, storage time limitations, maintenance of appropriate storage conditions, management of flows and vehicle capacity, and compliance with regulations.

### Proposed smart solution

After identifying the challenges related to MWM that require attention, we embark on exploring a wide array of technologies and smart solutions applicable to this field. Our aim is to consider emerging trends and innovations that have the potential to enhance current MWM practices. Additionally, we conduct an in-depth analysis of the perspectives of various stakeholders involved in MWM, including healthcare facilities, waste disposal agencies, warehouses, environmental experts, and regulatory authorities. This invaluable insight enables us to propose an approach that emphasizes fostering collaboration among all these stakeholders.

Based on the comprehensive problem analysis, literature review, and valuable inputs from stakeholders, we meticulously craft a detailed proposal for a smart solution tailored to MWM. Within this proposal, we outline the key components of the solution, such as incorporating smart containers, waste tracking systems, automation, and advanced treatment methods.

Moreover, we conduct a comparative study, pitting our proposed smart solution against the existing MWM process. This enables us to highlight the potential benefits our solution offers, including improved efficiency, cost savings, positive environmental impact, and enhanced compliance with relevant regulations. The comparative analysis showcases the advantages that our smart solution brings to the table, reaffirming its potential to revolutionize MWM practices.

### XAI dashboard for MWM

One of the key contributions of our article is the proposal of XAI solution for MWM. To achieve this the methodology used is as follows:

*Problem identification*

We begin by identifying the challenges in MWM and recognizing the lack of transparency in AI models. This step is essential to understand the significance of XAI in enhancing end-user confidence.

*Medical waste bin filling prediction:*

The case chosen for XAI implementation is the AI model for predicting the filling level of medical waste bins. By accurately predicting the filling level of medical waste bins, healthcare facilities can plan waste collections more efficiently, optimizing the use of resources such as transportation and personnel. ^55^ This can lead to cost savings and reduce the environmental footprint of waste management processes. Additionally, the proper management of medical waste is essential to prevent public health risks. If waste bins are not collected frequently enough and overflow, it can lead to contamination and the spread of diseases. On the other hand, if bins are collected too often, it can result in excessive fuel use and high collection costs. The AI model helps strike the right balance and ensures that waste disposal is timely and efficient. Moreover, medical waste can contain hazardous materials that can be harmful to both humans and the environment if not handled properly. By accurately predicting the filling level of waste bins, healthcare facilities can better manage the disposal of hazardous waste, reducing the risk of environmental contamination.

The data used in our study is based on declarations from the WHO, which provides reliable information on the average quantity of hazardous waste generated per hospital bed per day (0.5 kg per bed per day). ^56^ This information helps us model the waste generation accurately and create a predictive model that can assist in optimizing waste management practices.

We specifically focused on hospitals in the Casablanca region of Morocco as examples to demonstrate the effectiveness of our AI model in a real-world scenario. The availability of such data ^57^ allows us to develop and test our XAI solution, ensuring its relevance and applicability to MWM in this specific region.

*XAI library selection*

The solution employs various Python libraries for enhancing the XAI model’s functionality ^57^:

Scikit-learn [RRID:SCR_002577] is instrumental in our XAI implementation, serving multiple essential functions. Firstly, we use it for data preprocessing, handling data cleaning, feature scaling, and imputing missing values in the dataset. Secondly, we employ its feature selection technique, Recursive Feature Elimination (RFE), to identify the most relevant features that significantly contribute to the prediction task. Lastly, scikit-learn is used for model training, where we utilize Decision trees algorithm to create a tree-like model that makes decisions based on feature values at different nodes. Additionally, we leverage random forests to combine multiple decision trees, leading to more accurate predictions and reducing overfitting.

The main focus of our XAI solution is on model interpretability, which is why we opt for Decision trees due to their hierarchical structure. This enables clear understanding of decision-making at each node based on feature values, making them ideal for our XAI solution. This approach empowers stakeholders to comprehend the factors influencing predictions in MWM.

In summary, scikit-learn’s data preprocessing capabilities enable us to handle data cleaning and feature scaling, while its feature selection techniques help us identify the most relevant features. The use of decision trees and random forests ensures we build interpretable and accurate models for predicting medical waste bin filling levels, providing transparent and reliable insights for waste management.

Furthermore, we enhance our XAI model’s performance and accuracy by incorporating xgboost [RRID:SCR_021361], catboost [RRID:SCR_021694], and lightgbm [RRID:SCR_021697] libraries. Xgboost is employed to build multiple weak learners (decision trees) and combine them into a strong predictive model. This boosting technique corrects errors from previous models, improving predictive performance. By integrating Xgboost with decision trees and random forests, we achieve a balance between interpretability and accuracy in our XAI solution, maintaining transparency and reliability while accurately predicting medical waste bin filling levels.

To tackle the challenges of categorical feature handling in waste management data, we turn to Catboost. ^57^ In this domain, data often contains categorical variables like types of waste, hospital locations, or waste disposal methods, which require numerical representations for modeling. Catboost’s categorical feature support and advanced optimization techniques address this issue, enhancing accuracy and interpretability in our XAI model.

In the MWM process, data involves diverse features and observations from various healthcare facilities. Lightgbm’s “leaf-wise” tree growth strategy allows it to create deeper and more complex trees compared to traditional approaches, capturing intricate relationships within the data effectively. Moreover, the histogram-based binning reduces memory usage and speeds up computations, making Lightgbm efficient for processing vast amounts of waste management data while maintaining model interpretability.

By incorporating these high-performance gradient boosting libraries (Xgboost, Catboost, Lightgbm), our XAI model ensures accurate and reliable predictions for MWM tasks.

Throughout development, we prioritize transparency and reproducibility by using specific version numbers for each library, including scikit-learn (v0.16.1), ^58^ xgboost (v1.7.6), ^59^ catboost (v1.2) ^60^ and lightgbm (v4.0.0). ^61^ Adhering to these version numbers guarantees consistency and facilitates easy replication for future studies or real-world applications.

*Interactive dashboard*

Our XAI model generates an interactive dashboard that explains the inner workings of each deployed machine learning model. This dashboard presents the results of the following techniques.

The feature importance analysis technique provides insights into the significance of each input feature in the AI model’s predictions. This process is vital to understand which features (distance between hospitals, hospital size, vehicle capacity, and distance between hospital and warehouse) have the most substantial influence on the model’s performance and how they contribute to the predictions.

To calculate feature importance, we utilize decision trees and random forests. These algorithms assign importance scores to each feature based on their ability to split the data and make accurate predictions.

This process helps us identify the key variables that significantly impact the filling level of medical waste bins, enhancing the interpretability and efficiency of our AI model. We gain a clear understanding of which features drive the predictions and can make informed decisions in waste management strategies.

Additionally, we incorporate the SHAP (SHapley Additive exPlanations) approach to further enhance the interpretability of our machine learning model, which includes decision trees and random forests. The SHAP values, based on cooperative game theory, attribute the contribution of each feature to the model’s prediction for a specific sample. This empowers us to discern which features have the most significant impact on the filling level of medical waste bins. For example, if the model predicts a higher filling level for a particular medical waste bin, SHAP values help us understand which features contributed positively to this prediction and which features had a negative impact. This knowledge enables stakeholders in MWM to identify critical factors influencing predictions and make informed decisions to optimize waste collection, resource allocation, and waste management practices.

By leveraging the SHAP approach with decision trees and random forests, we gain a comprehensive understanding of the contribution of each feature to individual predictions for medical waste bin filling levels. This knowledge is essential for stakeholders in waste management to comprehend the factors influencing predictions and make informed decisions to optimize waste collection and resource allocation.

Furthermore, we incorporate the *confusion matrix* and performance metrics as essential components of our interactive dashboard, to evaluate the performance of our machine learning model, particularly for classification tasks, such as predicting whether a medical waste bin will reach a certain filling level or not.

The confusion matrix is a table that presents a detailed breakdown of the model’s predictions compared to the actual ground truth. It consists of four components:

• True positive (TP): The number of correct predictions made by the model for waste bins that are actually filled to the expected level. In the context of MWM, this means the bins that are correctly identified as being filled to the appropriate capacity.
• False negative (FN): The number of instances where the model predicted the bins to be not filled to the expected level, but in reality, they were filled. In MWM, FN could be critical, as they may lead to missing hazardous waste situations, potentially causing environmental and health risks.
• False positive (FP): The number of instances where the model predicted the bins to be filled to the expected level, but they were not actually filled. In our case study, FP could lead to unnecessary waste collection efforts and resource wastage.
• True negative (TN): The number of correct predictions made by the model for bins that are not filled to the expected level. These are bins that the model correctly identifies as not requiring immediate attention.

Using the values from the confusion matrix, we can calculate various performance metrics:

• Accuracy: It measures the overall correctness of the model’s predictions. Higher accuracy indicates a more reliable model.
$$\text{Accuracy}=\frac{\mathrm{TP}+\mathrm{TN}}{\mathrm{TP}+\mathrm{FP}+\mathrm{TN}+\mathrm{FN}}$$ (1)
• Precision: Also known as positive predictive value (PPV), it indicates the model’s ability to correctly identify positive instances.
$$\text{Precision}=\frac{\mathrm{TP}}{\mathrm{TP}+\mathrm{FP}}$$ (2)
• Recall: Also known as sensitivity or true positive rate (TPR), it assesses the model’s ability to correctly identify positive instances among all actual positive instances.
$$\text{Recall}=\frac{\mathrm{TP}}{\mathrm{TP}+\mathrm{FN}}$$ (3)

In the context of hazardous waste management, high recall is of paramount importance to avoid FN. FN represent cases where the model fails to detect potentially hazardous situations, which can lead to adverse consequences. By ensuring high recall, our XAI solution aims to detect and address hazardous waste situations promptly, contributing to more effective waste management practices and minimizing potential environmental risks.

In summary, the confusion matrix and performance metrics in our XAI dashboard provide a comprehensive evaluation of our machine learning model’s performance. These metrics offer insights into the model’s ability to correctly classify positive and negative instances, enabling stakeholders in MWM to make informed decisions and optimize waste collection and disposal strategies effectively.

Moreover, we employ the *AUC-ROC curve* (area under the curve (AUC) of the receiver operating characteristic (ROC)), and *PR AUC curve* (area under the precision-recall), as essential evaluation measures to assess the performance of our AI model for predicting the filling level of waste bins.

• The ROC curve is a graphical representation that illustrates the model’s TP rate (recall) against the FP rate at various classification thresholds. We use this curve to illustrate the trade-off between sensitivity (correctly identifying filled bins) and specificity (correctly identifying non-filled bins). By plotting these rates, we can visualize how the model’s performance changes as we adjust the classification threshold.
• The AUC is a single numerical value that summarizes the performance of the ROC curve. It represents the area under the ROC curve, with a higher AUC indicating better discrimination between the two classes (filled and unfilled waste bins). A perfect classifier would have an AUC value of 1, while random guessing would result in an AUC of 0.5.
• We also consider the PR-AUC. This metric is particularly useful when dealing with imbalanced datasets, where one class (e.g., filled waste bins) is more prevalent than the other. The PR curve represents the trade-off between precision (positive predictive value) and recall (true positive rate) at different classification thresholds. It demonstrates our model’s ability to correctly identify positive instances while minimizing FP.

Additionally, we use the lift curve to assess the performance of our machine learning model in identifying the level of filling of waste bins. Indeed, this curve identifies how much better our model is at capturing positive instances compared to a random model.

To calculate the lift, we divide the percentage of filled bins correctly identified by the model at a given percentile by the overall percentage of filled bins in the dataset. This gives us a measure of how much our model is lifting the filled bins’ detection compared to random chance. We plot the lift curve using the lift values calculated at different percentile points. The x-axis represents different percentile points, while the y-axis represents the lift values. The curve shows how the lift changes as we move through the sorted predictions.

The lift curve analysis helps us identify the most suitable classification threshold for making predictions. It allows us to determine the point at which our model is most efficient in detecting filled bins, guiding our decision-making for waste management efforts. By utilizing the lift curve, we can prioritize the identification of filled bins accurately, reducing potential risks and environmental impact in MWM.

Furthermore, we utilize the contribution graph to represent the SHAP values, showing the impact of individual features on the model’s predictions. Positive and negative contributions indicate whether a feature increases or decreases the prediction, respectively. The contribution graph helps stakeholders easily grasp how changes in different features influence the model’s decision-making process.

We also use the partial dependency graph to demonstrate how the target variable’s prediction changes as a specific feature varies while holding other features constant. This provides insights into non-linear patterns and interactions between features, allowing us to gain a deeper understanding of how different features affect the model’s predictions in the context of medical waste bin filling levels.

In conclusion, the proposed XAI solution for MWM addresses transparency and interpretability issues in AI models. It leverages various libraries and evaluation measures to provide reliable and understandable predictions for medical waste bin fill levels. The interactive dashboard empowers stakeholders to make informed decisions and optimize waste management practices based on transparent and trustworthy insights from the XAI model.

### TDVRPTW with two sub-models for MWM

Our article presents also a solution for the TDVRPTW specifically tailored for MWM, the primary objective is to optimize the collection and transportation of medical waste while considering time constraints and ensuring efficient resource utilization. To achieve this, we propose a novel approach that involves the development and testing of two sub-models: one focused on waste collection process from various hospitals and the other dedicated to waste transportation to treatment centers.

*Problem description*

We begin by describing the problem for each sub-model. In the waste collection sub-model, we aim to find the most efficient schedules for waste pickup from different hospitals, considering constraints such as time windows, vehicle capacity limits, and known waste quantities. Similarly, in the waste transportation sub-model, our objective is to determine the optimal routes for transporting the collected waste to treatment centers while adhering to time windows and vehicle capacity constraints.

*Assumptions*

After describing the problem, we will define and justify the key assumptions made during the model development. These assumptions are essential to simplify the problem and enable a more manageable approach while still capturing important real-world considerations. Each assumption serves a specific purpose in the model, facilitating the optimization process and ensuring practicality in addressing MWM challenges. By acknowledging these assumptions, we can develop a comprehensive and efficient solution that lays the groundwork for further refinement and adaptation to real-world scenarios with more complex factors.

*Constraints and objective function*

We will define the constraints and the objective function for each sub-model based on the problem descriptions. These constraints are crucial for ensuring the practicality and feasibility of the proposed solution. By incorporating constraints such as respecting time windows for waste pickup and delivery, vehicle capacity limitations, and known waste quantities, the model can effectively address real-world operational considerations. Additionally, the objective function will be formulated to minimize the traveling cost, taking into account various factors such as distance, time, and resource utilization. This objective function aligns with the goal of optimizing waste collection and transportation processes to reduce costs and enhance overall efficiency.

*Mathematical model*

With the constraints and objective function defined, we construct the mathematical model for both sub-models. This model represents the optimization problem in a mathematical framework, allowing us to apply optimization algorithms to find optimal solutions efficiently.

*Algorithm used*

**Figure 2.**
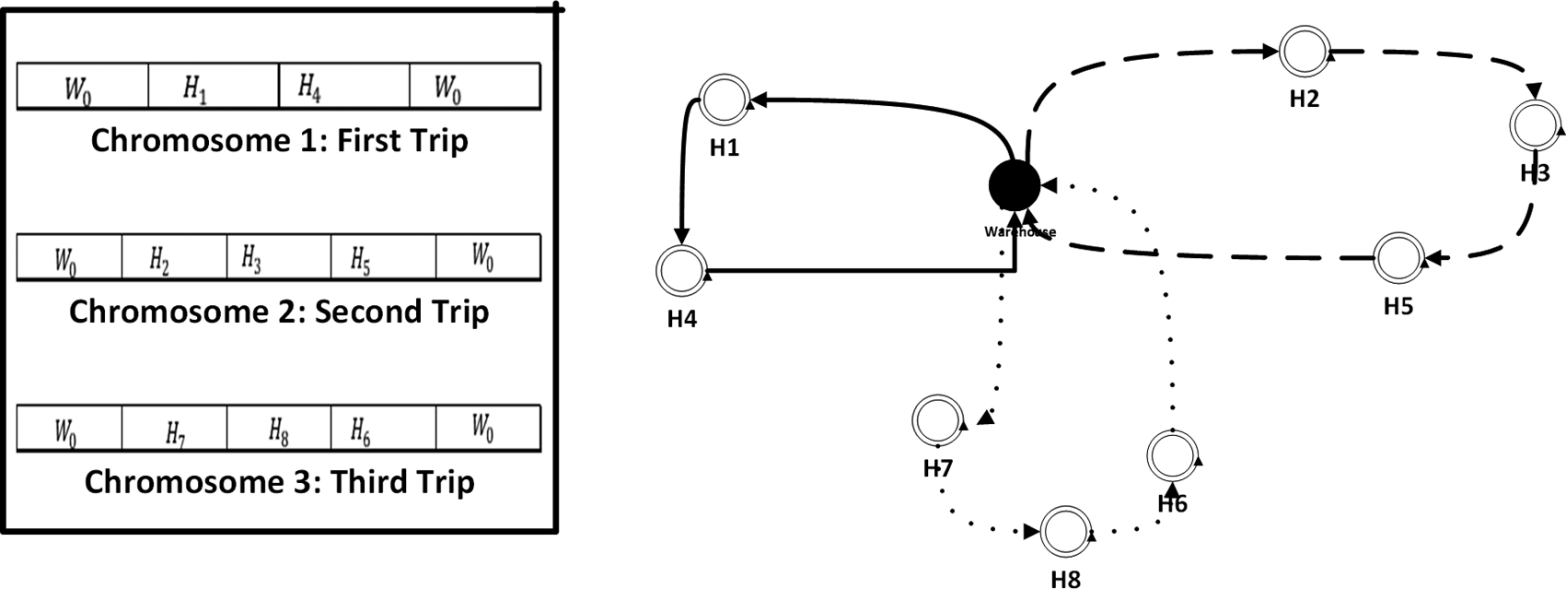
Representation of chromosome.

To address the TDVRPTW in both sub-models of MWM, we employ a genetic algorithm (GA) due to its effectiveness in exploring complex solution spaces and finding near-optimal solutions. The GA follows essential steps:

• *Chromosome definition*: The definition of chromosomes is a crucial step in the GA, as it determines the set of possible solutions that the algorithm will consider. In our case “optimizing the collection of medical waste”, a chromosome represents a possible schedule for waste collection, including the order in which different nodes are visited, the routes taken, and the quantities of waste collected at each hospital (see Figure 2).
• *Initialization*: The initialization is the first step in the GA, in our case it is generated randomly of 100 chromosomes. Each of these chromosomes is evaluated and assigned a fitness score based on how well it solves the problem (In term travelling cost of collecting medical waste). The best-performing solutions are then selected for reproduction. Indeed, we use the tournament selection to select a subset of schedules from the population, and evaluating each schedule based on its fitness score (the objective function). The schedule with the highest fitness score in that subset is then selected as a parent for the next generation.
• *Crossover*: Selected chromosomes undergo crossover using the random sequence insertion based crossover method (RSIX). The RSIX is a variation of the single-point crossover method that is designed to preserve the order of genes in the chromosome. In our case “optimizing the collection of medical waste”, this crossover method might be used to combine schedules from two parent solutions to create a new schedule for the next generation while preserving the order of the sites to be collected.

The RSIX operates in the following manner:

1. Choose two parent solutions from the current population.
2. Select a random position along the chromosome of one of the parents.
3. Choose a random subset of the genes (i.e., collection sites) to the right of the selected position in the first parent.
4. Insert the randomly selected subset of genes into the same position in the second parent, maintaining the order of the genes in the second parent.
5. Remove any duplicates that may have resulted from the insertion process.
6. The resulting offspring is tested for fitness value and then added to the next generation population.

• *Mutation:* The next step in the GA after the crossover operation is the mutation operation. The mutation involves randomly changing two sites in a given schedule to a different time to improve chromosome. This step is used to explore new regions of the solution space and to prevent premature convergence to a suboptimal solution.
• *Iterative process:* The selection, crossover, and mutation steps are iteratively performed across multiple generations until a stopping criterion is met. The criterion could be reaching a maximum number of generations or achieving a satisfactory solution. The best candidate solution with the lowest traveling cost is selected as the optimal schedule for waste collection and transportation.

In this study, our primary focus is on proposing a comprehensive TDVRPTW model that involves three entities: hospitals, warehouses, and treatment centers. While we do not contribute a novel algorithm, we implement a GA from an existing paper. ^62^ The GA is designed to handle multiple vehicles with varying capacities and travel times between different nodes, making it well-suited for tackling complex vehicle routing scenarios.

To evaluate the performance of the algorithm used, we conducted simulations using different sets of parameters. Moreover, we conduct a performance comparison with another existing algorithm for TDVRPTW, developed by Ref. 63. This comparison is carried out using the ANOVA (analysis of variance) test and Tukey post-hoc analysis. These statistical tests are performed using Minitab software (RRID:SCR_014483) (version 18.1), ^64^ and the results can also be obtained using Python or R. The aim of these tests is to identify any significant differences in the distances traveled by the algorithms in MWM scenarios.

*TDVRPTW simulation*

The proposed method for evaluating the TDVRPTW model involves conducting simulations with different parameter sets. The simulations are performed using a GA to find solutions for the TDVRPTW instances. The model’s performance is assessed based on various metrics, such as the total distance traveled by vehicles, the total travel time, the number of trucks used, and the simulation runtime. The effectiveness and robustness of the GA are evaluated by considering variations in cluster structures, random aspects, and scheduling horizons. The algorithm’s adaptability to diverse conditions and its efficiency in resolving vehicle routing problems with time windows are also studied, with a particular focus on handling instance diversity and addressing real-world time constraints and operational conditions.

Parameter sets: Each parameter set includes values for various parameters that influence the behavior of the GA used to solve the TDVRPTW model. The parameters include:

• ts_prob: Probability of applying time-dependent search operators
• x_prob: Probability of applying crossover
• m_prob: Probability of applying mutation
• w_t: Time window penalty factor
• mng: Maximum number of generations
• pop_size: Population size
• init_method: Initialization method (e.g., random sample)
• cache_gran: Cache granularity

Instances: The instances represent specific scenarios of the TDVRPTW model, each denoted by abbreviations ( *e.g.,* C101, C102, R101, RC101, *etc.*). For each instance, the simulation results provide the following metrics:

• Score: A measure of efficiency or optimization quality obtained for that instance.
• Distance: Total distance traveled by the vehicles in the solution.
• Travel Time: Total travel time for the vehicles in the solution.
• Runtime (Sec): The time taken for the GA to complete the simulation.
• Vehicles: The number of vehicles used in the optimized solution for that instance.

Simulation results: For each instance, the simulation results are presented side by side, comparing two different parameter sets’ performance. The results highlight the performance of the GA in finding solutions for the TDVRPTW under various parameter combinations. The goal is to assess how different parameter settings affect the efficiency and quality of the solutions produced by the algorithm.

By conducting these simulations and analyzing the results for different instances and parameter sets, the effectiveness, adaptability, and robustness of the TDVRPTW model can be evaluated.

*Statistical tests*

*ANOVA Test*

The ANOVA test is used to analyze the variation observed between the means of two or more groups. Its primary purpose is to test the hypothesis of whether these group means are equal or not. In our study, we are interested in comparing the performance of two algorithms: the algorithm utilized in this specific research ^62^ and a widely adopted TDVRPTW algorithm commonly used in practice. ^63^ The comparison will be based on the distance traveled across six distinct classes. The ANOVA model can be written as:

$$Y_{\mathrm{ij}}=\mu +A_{i}+B_{j}+\epsilon_{\mathrm{ij}}$$ (4)

Where $Y_{\mathrm{ij}}$ represents the observed response of the jth observation in the ith treatment group, μ is the overall mean, $A_{i}$ is the effect of the ith level of factor A (problem class), $B_{j}$ is the effect of the jth level of factor B (algorithm used), and $\epsilon_{\mathrm{ij}}$ is the random error.

The null hypothesis is that there is no difference between the means of the groups, which can be written as H0:μ1=μ2=…=μk . The alternative hypothesis is that at least one group mean is different from the others.

The ANOVA test is based on three main assumptions:

• Normality: The distribution of the errors should be normal (conditional residual value charts are used to check the assumptions of normality and homoscedasticity in the ANOVA model.
• Homogeneity of variance: The variance of the errors should be equal across all groups.
• Independence: The observations should be independent of each other.

If these assumptions are met, we can use the F-test to determine if there are significant differences between the means of the groups. The F-statistic is calculated as the ratio of the between-group variance to the within-group variance, and follows an F-distribution with k-1 and n-k degrees of freedom, where k is the number of groups and n is the total sample size. When the computed F-value exceeds the critical value, we reject the null hypothesis and infer that there exists a noteworthy distinction between the means of the groups.

*Tukey test*

In the study, we analyze the performance of two algorithms for TDVRPTW: the “GA used” algorithm ^62^ and the ALNS algorithm. ^63^ To conduct a comprehensive comparison, we divide the problem instances into six distinct classes, each representing a different scenario:

C1: Clustered instances with a short scheduling horizon.

C2: Clustered instances with a long scheduling horizon.

R1: Random instances with a short scheduling horizon.

R2: Random instances with a long scheduling horizon.

RC1: Random and clustered instances with a short scheduling horizon.

RC2: Random and clustered instances with a long scheduling horizon.

These classes are formed based on various characteristics of the problem instances, such as the spatial distribution of hospitals (clustered or random) and the time horizon for scheduling (short or long). By categorizing the problem instances into these classes, we can assess how each algorithm performs under different conditions and gain insights into their strengths and limitations.

After conducting the ANOVA test to determine if there are overall significant differences in the algorithms’ performance, we employ the Tukey test as a post-hoc analysis. The Tukey test allows us to perform specific pairwise comparisons between the means of each class for both algorithms. By doing so, we can identify which classes exhibit significant differences in terms of distance traveled for each algorithm. This in-depth analysis helps us understand how the algorithms fare in different problem scenarios and enables us to make informed decisions about their suitability for solving real-world MWM problems.

For statistical analysis, a significance level of 0.05 is set to determine statistical significance, ensuring that any observed differences in algorithm performance are reliable and not merely due to chance.

By conducting these rigorous statistical tests and comparing the algorithms’ performance across different problem instances, we can confidently recommend the most effective algorithm for optimizing medical waste transportation, ultimately contributing to efficient and sustainable waste management practices in healthcare facilities.

*Data definition*

To assess the effectiveness of our proposed model, we employ two distinct datasets for testing and validation. The first dataset, known as the Synthetic Dataset (Solomon Instances), comprises benchmark instances commonly used for testing vehicle routing problems. These benchmark instances are well-established and can be referenced from academic sources and previous research in the field of vehicle routing optimization. ^65^

On the other hand, the second dataset, referred to as the Real-World Dataset (Average Medical Waste Generation per Bed), ^57^ is based on declarations from the WHO. The WHO provides reliable information on the average quantity of hazardous waste generated per hospital bed per day, allowing us to accurately model waste generation and create a predictive model to optimize waste management practices. It is important to note that the data from the WHO provides valuable insights into real-world medical waste generation scenarios, making our model more practical and applicable to healthcare settings.

For this study, we specifically focused on hospitals in the Casablanca region of Morocco, taking into account their unique characteristics and waste generation patterns. By tailoring our approach to this specific region, we can address the particular challenges and requirements of MWM in Casablanca and provide targeted solutions for enhancing waste collection and transportation processes in the area.

*Problem resolution using GA*

Finally, we apply the GA to both sub-models to find the most optimal solutions for waste collection and transportation. The GA iteratively explores different schedules and routes, considering the defined constraints and objective function, until satisfactory solutions are obtained.

By following these steps for both sub-models, we ensure a systematic and comprehensive approach to optimizing MWM operations, leading to efficient waste collection and transportation while minimizing associated costs and meeting the specified constraints.

### Optimizing MWM through cross-docking: A simulation-based study in the Casablanca region

The objective of this study was to illustrate the benefits of a warehouse for cross-docking in MWM and evaluate its impact on various waste management strategies. To achieve this, we utilized a simulation modeling approach implemented in Simul8 (v 3.0) ^66^ (For an open-source alternative it may be possible to use SimPy). The simulation aimed to replicate real-world MWM scenarios while thoroughly testing the influence of cross-docking with waste sorting.

To ensure the reliability of the simulation, we conducted a significant number of runs, totaling 100 runs for two waste management scenarios. The first scenario involved waste distribution without prior sorting, where medical waste was directly transported from hospitals to treatment centers without any intermediate sorting process. The second scenario involved setting up a warehouse for cross-docking with waste sorting, where medical waste from hospitals was first transported to a centralized cross-docking center, sorted according to its treatment type, and then sent to specialized processing centers accordingly. By running multiple simulations for each of these two scenarios, we were able to gather substantial data and effectively assess the system’s behavior under various conditions. Considering multiple runs allowed us to reduce the impact of random variations and enhance the overall reliability of the simulation results for both waste management strategies.

Throughout the simulation runs, we meticulously recorded and analyzed various performance indicators, such as processing costs, processing times, transport costs, environmental impacts, and initial investment costs. This extensive data collection facilitated a thorough comparative analysis between the two waste management strategies: waste distribution without sorting and cross-docking with waste sorting.

Data used in our simulation:

Number of medical waste produced per day: 500 kg

Percentage of recyclable waste: 40%

Number of workers needed to sort waste: 3

Hourly cost of a worker: €20

Cost per kilogram of waste treatment: €0.50

Initial investment cost for setting up the cross-docking warehouse: €50,000

Transport cost per kilometer: €1/km

Distance between the waste production center and processing center: 20 km

Processing time per kilogram of waste: 2 minutes

Cross-contamination rate in the case of distribution without sorting: 25%

Cross-contamination rate in the case of cross-docking warehouse with sorting: 5%

In this simulation, we focused on the Casablanca region of Morocco in terms of size and waste management practices.

By conducting this simulation and thoroughly analyzing the performance indicators, we aimed to provide valuable insights into the advantages of cross-docking with waste sorting and make data-driven decisions to optimize MWM practices in the Casablanca region. The simulation results provided comprehensive information on processing costs, processing times, transport costs, and environmental impacts, enabling us to identify the most efficient waste management strategies for healthcare centers and hospitals in the region.

## Proposed solution

### Smart MWM system

**Figure 3.**
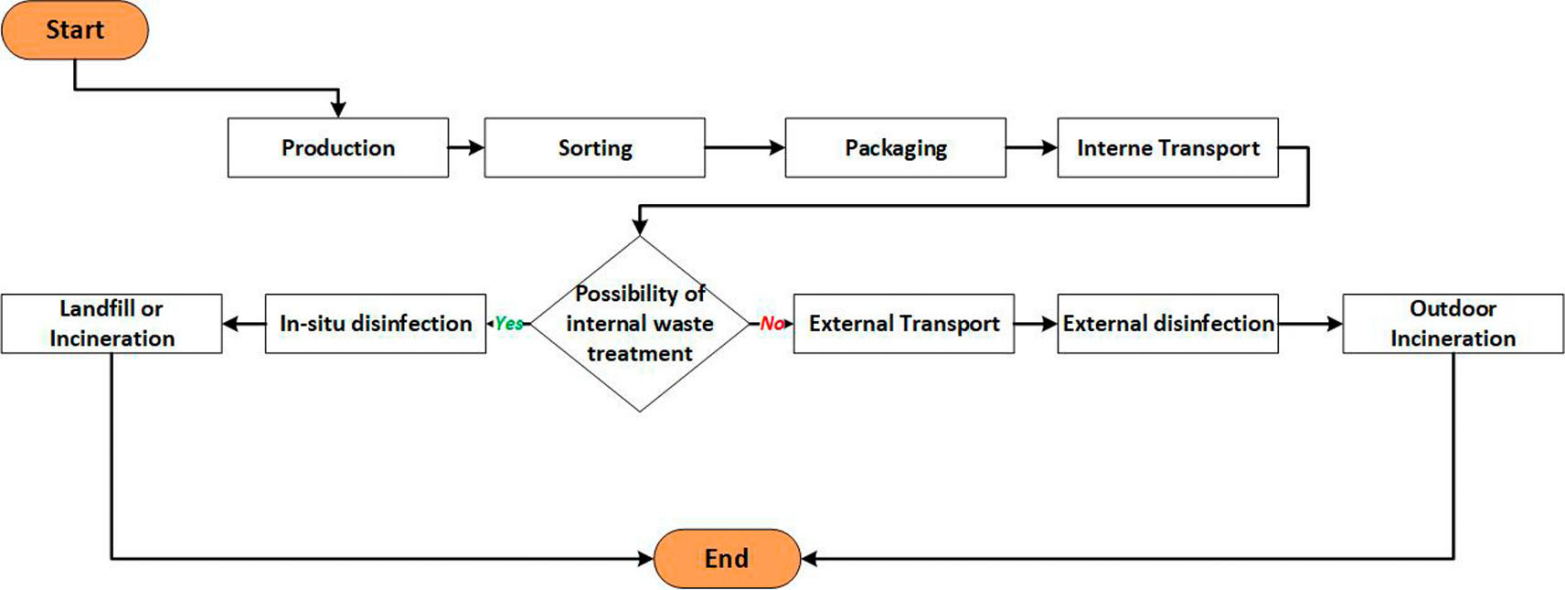
The current MWM process.

The MWM process involves several essential steps: sorting and packaging, storage, transport, treatment, and disposal, as depicted in Figure 3. ^6^ However, this process faces numerous challenges, including the need to address health risks, adhere to storage time limits, and maintain appropriate storage temperatures. It is also crucial to manage waste flows, optimize vehicle capacity in relation to waste generation, and mitigate risks during transportation. Moreover, strict compliance with regulations, cost control of outsourcing services, and minimizing environmental impact are key considerations at the treatment unit.

**Figure 4.**
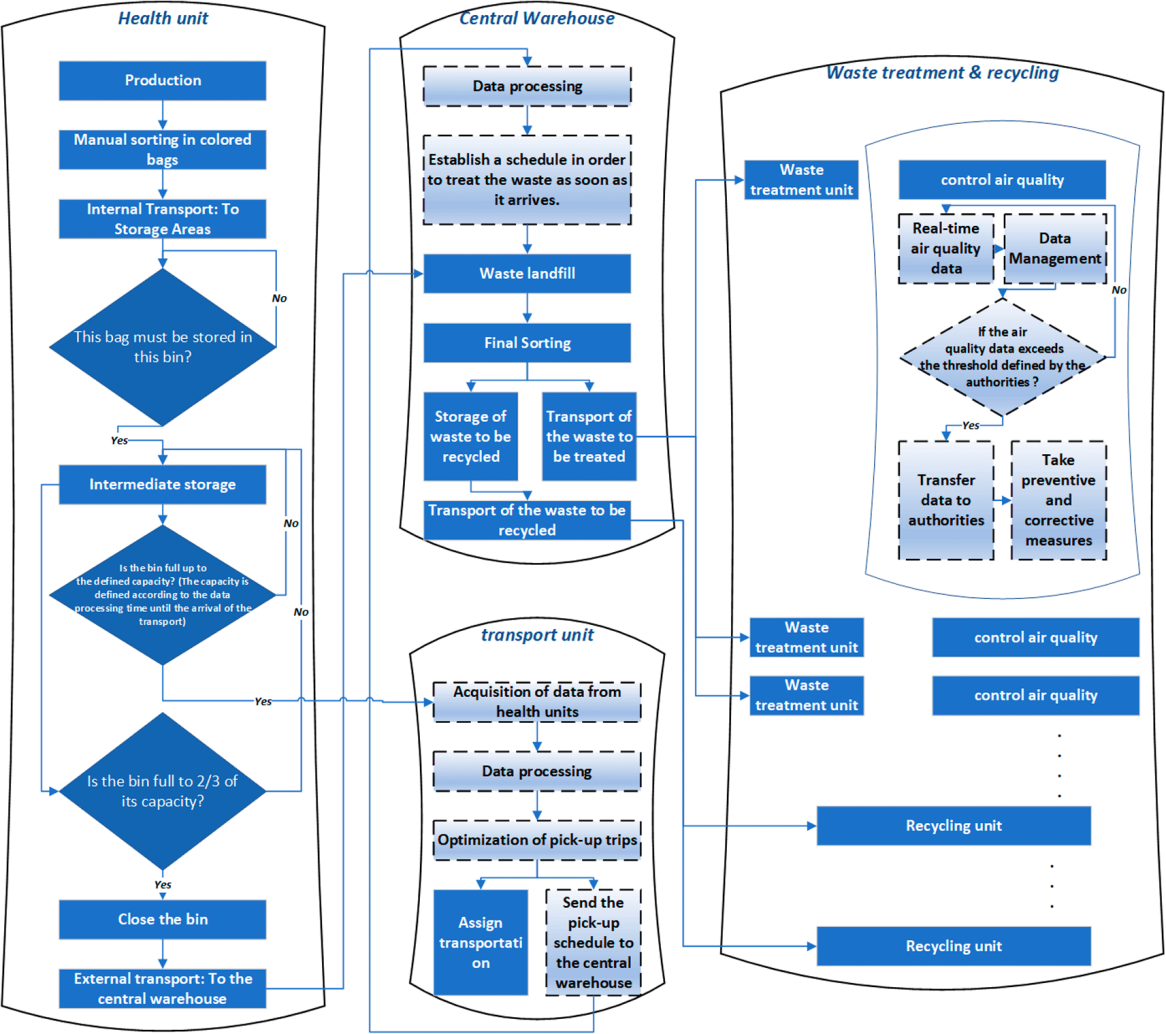
The proposed smart medical waste management system.

To address these challenges effectively, we propose a smart solution that promotes collaboration among all stakeholders, as illustrated in Figure 4. Our proposed process begins with the initial sorting of medical waste into colored bags, each equipped with an identification tag. The sorted waste is then transported to designated secure sites. At these sites, specialized smart containers are utilized for storing each type of waste, ensuring proper segregation and management.

The smart containers used in our proposed solution are equipped with advanced sensors and actuators to perform various essential functions. These include humidity and temperature sensors to monitor storage conditions, a level sensor to prevent exceeding the maximum storage capacity, an actuator for automated container closure after filling, and an object recognition sensor to ensure proper sorting compliance. When a container reaches its defined maximum capacity (determined based on transport arrival time and filling frequency), a notification is sent to the transport unit to arrange for timely collection. Additionally, if a container reaches two-thirds of its capacity before the transport’s arrival, it will automatically close.

Upon the transport’s arrival, the containers are sent to the warehouse for final sorting. This stage involves identifying waste suitable for recycling and waste that requires appropriate disposal (with further sorting based on the specific disposal methods). The warehouse sorting process employs intelligent conveyor belts integrated with object recognition sensors.

Subsequently, the waste is reloaded and transported to designated treatment units based on the recommended treatment type. Throughout the treatment operations, adherence to regulations and environmental considerations are crucial. To ensure compliance, we propose the implementation of real-time air and soil control sensors, generating data to inform decision-making and enforce necessary measures.

In our approach, we have opted to outsource the entire waste treatment process, enabling specialized units to focus on this critical task while allowing hospitals to concentrate on providing essential healthcare services. This centralization of waste treatment helps minimize the environmental impact by confining it to specific areas.

To maintain appropriate transport conditions, vehicles are equipped with a GPS and identification system to track waste during transportation. Additionally, humidity and temperature sensors are installed to monitor and maintain optimal conditions throughout the transport process.

The current and proposed solutions follow the same basic process of sorting, storing, transporting and treating/disposing of medical waste. However, the proposed solution uses a more sophisticated and technologically advanced approach to ensure proper management of medical waste (see Table 1). The proposed solution includes the use of different colored bags equipped with tags to facilitate the sorting of medical waste, as well as smart containers equipped with sensors to monitor the storage conditions and capacity of each container. In addition, we use object recognition sensors to help sort the waste and direct it to the appropriate treatment units. The proposed solution also includes outsourcing waste processing to allow hospitals to focus on core healthcare services. Air and soil monitoring sensors are used to ensure that environmental regulations are met and that medical waste processing is done in a safe and responsible manner. In conclusion, the proposed solution is more technologically advanced and allows for more efficient and safe MWM while complying with environmental regulations.

**Table 1.** A comparative study: evaluating the current solution versus the proposed solution.

Steps	Current solution (Moroccan context) ^6^	Proposed solution
Sorting	Manual sorting of waste	Use of different colored bags equipped with tags and object recognition sensors and Smart Warehouse for final sorting according to the type of treatment needed
Storage	Regular containers	Smart containers with sensors
Transport	Conventional methods	Use temperature and humidity sensors and a GPS and identification system
Treatment/disposal	Internal and external processing	Outsourcing with environmental monitoring sensors

As discussed in the previous section, a smart MWM system can improve the efficiency and safety of waste management. However, the management of medical waste is a complex process that involves multiple stakeholders and requires real-time monitoring and decision-making. This is where an XAI dashboard can be useful. By integrating AI and machine learning algorithms, an XAI dashboard can provide hospitals and waste management companies with real-time insights and data analytics to help them make better decisions and optimize their waste management processes.

### XAI dashboard for MWM

AI models are widely used effectively for different applications, however these models lack transparency due to the black box mechanism of AI. In order to gain end-user confidence in AI applications, they must be supported by reliable and unbiased decision results or convincing rationalization and justification, which is the role of XAI.

In our solution, we use the XAI library ^67^ to enable the relevant stakeholders to analyze the end-to-end solution and identify discrepancies that may result in sub-optimal performance with respect to the required objectives. More generally, the proposed XAI model is designed based on three steps: data analysis, model evaluation and production monitoring.

**Figure 5.**
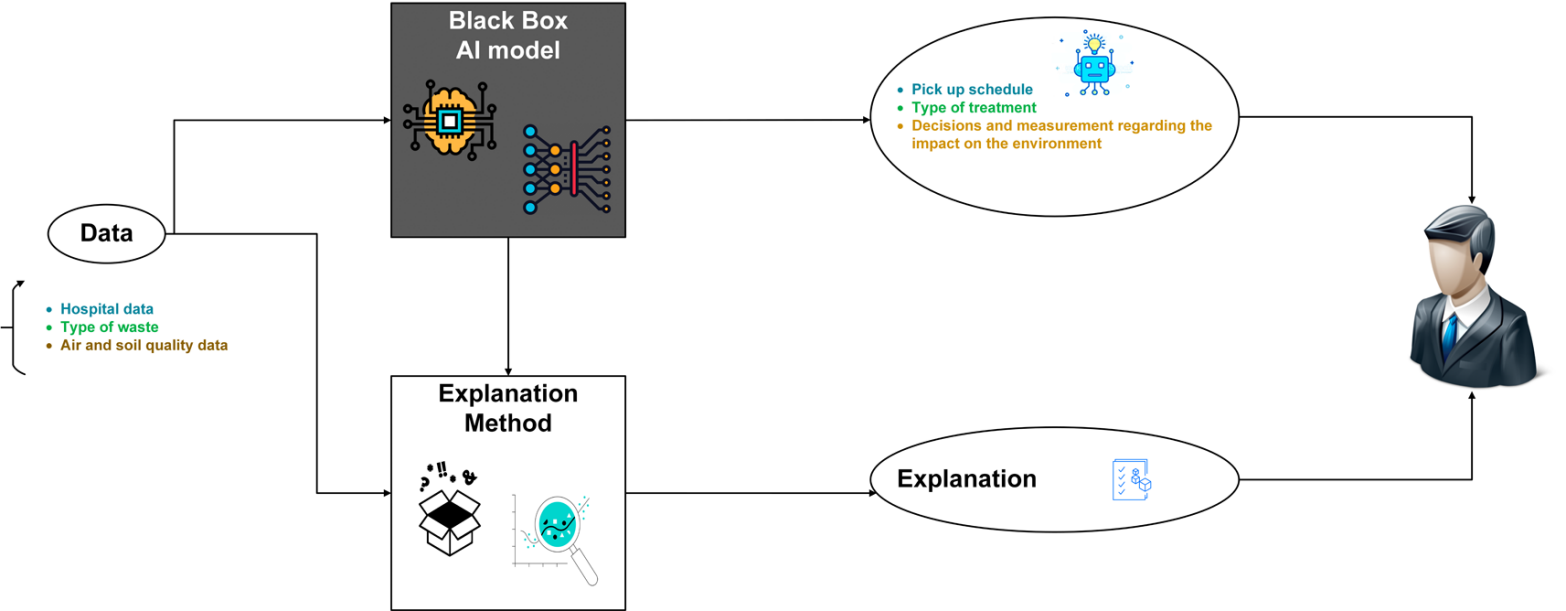
Explainable artificial intelligence (XAI) in medical waste management system.

Figure 5 provides a visual overview of XAI in MWM.

The case that we will approach for XAI implementation is the AI model for the prediction of the filling level of medical waste bins. We have chosen this component because of its importance for their management as it allows to plan the collections in a more efficient way and to optimize the use of the resources. If bins are collected too often, it can lead to excessive fuel use and high collection costs. Conversely, if bins are not collected often enough, they may overflow, which can lead to public health risks and additional costs for cleaning and disinfection. Forecasting the fill level of medical waste bins also allows for better management of human resources by assigning collection tasks at the most efficient times and avoiding unnecessary wait times. Finally, better management of medical waste bins can help reduce environmental impact by minimizing the amount of waste transported and optimizing collection routes.

Our solution is designed with a strong focus on interpretability, ensuring that it is not limited to data scientists but also accessible and understandable to end users, indeed the decisions and predictions generated by our XAI model are presented in a manner that can be easily understood and trusted by individuals who may not have a deep understanding of the underlying machine learning algorithms.

We prioritize the transparency of our model’s decision-making process, enabling users to comprehend the factors influencing the outcomes and fostering trust in the system.

We propose an interactive dashboard that explains the inner workings of each deployed machine learning model. This allows us to open the “black box” and show users, stakeholders, how our smart system generates its predictions. This dashboard includes:

• “Feature importances” to explain the selection of appropriate features (distance, hospital size, vehicle capacity, and distance between hospital and warehouse) of the AI model,
• “SHAP” approach to explain the output of our machine learning model.
• “Confusion Matrix, Lift Curve and Cumulative Accuracy” to compare the actual target values with those predicted by our machine learning model (bin fill status), which gives us an overview of the performance of our classification model and the types of errors it makes,
• Analyze the prediction of each node (Hospital), and explain how each feature contributed to this prediction “Contribution Graph”, and how this prediction changes if we change a feature “Partial Dependency Graph”.
• “Feature dependencies” to identify the relationship between the feature value and the SHAP value
• “Decision trees” inside a random forest.

**Figure 6.**
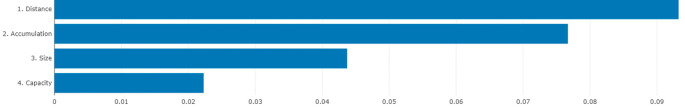
Average impact on bin fill predictions (average absolute value of SHapley Additive exPlanations (SHAP)).

To predict the level of filling of bins in hospitals, it is essential to take into account specific features that will have a larger effect on our learning model. To do this, our XAI model uses feature importance techniques that compute a score for all input features, these scores simply explain the importance of each feature (see Figure 6).

The feature importance allows the end user and stakeholders to understand the relationship between the features (Distance, Hospital Size, Capacity of vehicle and Accumulation rate) and the target variable (Filling of bins), also identify which features are irrelevant for our model. In addition, the highest scores are usually kept and the lowest scores are ignored as they are not important for the model. This not only simplifies the model, but also speeds up its execution, which ultimately improves the performance of the model. In our case, the vehicle capacity is fixed regardless of the hospital and does not affect the performance of our algorithm.

**Figure 7.**
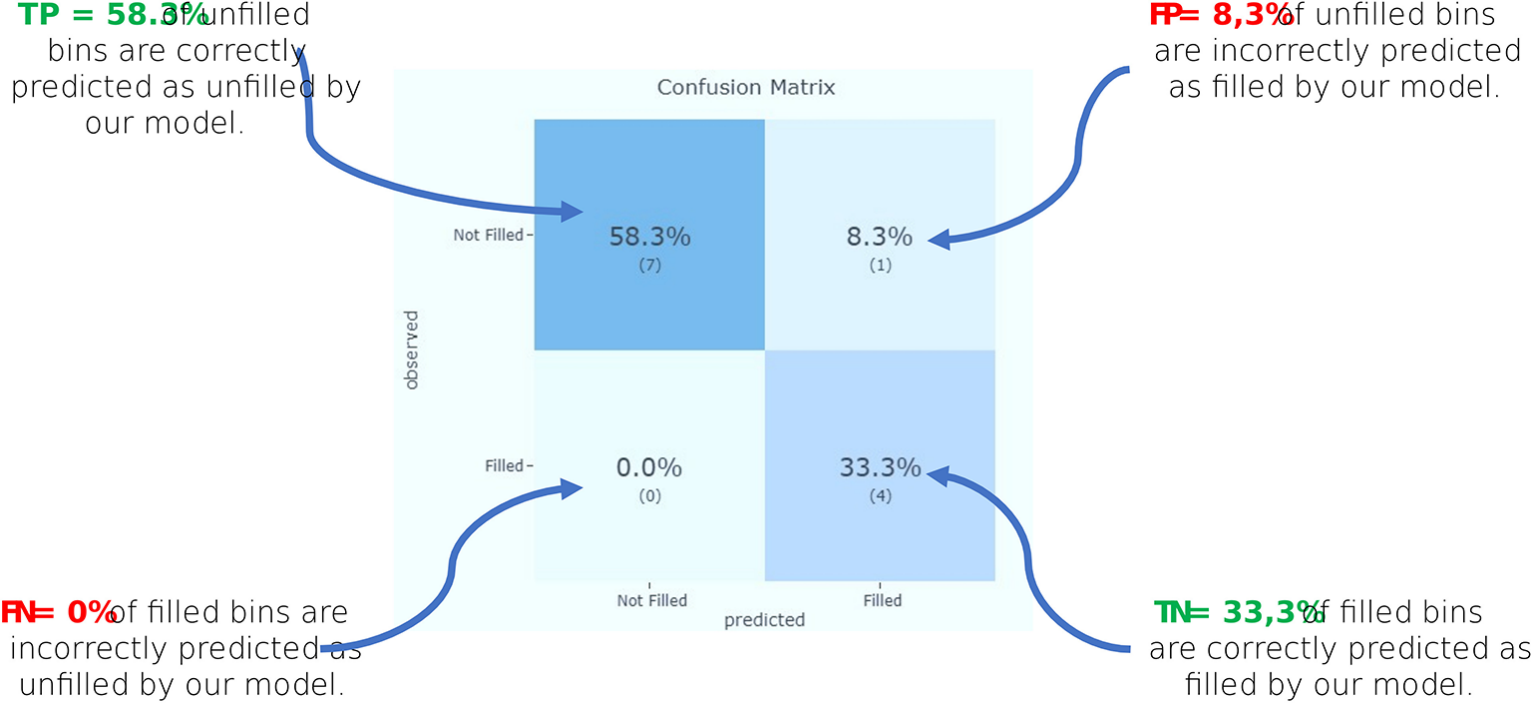
Confusion matrix.

To compare the target values with those predicted by our machine learning model, we implement a confusion matrix in our dashboard. This gives us a holistic view of how well our prediction model is performing and what kinds of errors it is making (see Figure 7). From the confusion matrix, we identify the accuracy of our model, as well as the precision and recall.

According to these results (see Table 2), we can see that “Precision” 80% of the correctly predicted cases actually turned out to be positive. Whereas “Recall” 100% of the positives were successfully predicted by our model.

**Table 2.** Prediction model performance metrics.

Model performance metrics	
metric	Score
Accuracy	**0.917**
Precision	**0.8**
Recall	**1**
f1	**0.889**
roc_auc_score	**1**
pr_auc_score	**1**
log_loss	**0.394**

Recall is important in MWM (especially hazardous waste) where it is unimportant to generate a false alert, but actual positive cases should not go unnoticed. In our example, recall would be a better measure than precision, because we don’t want to increase the risk of hazardous waste, which would spread contagious germs.

**Figure 8.**
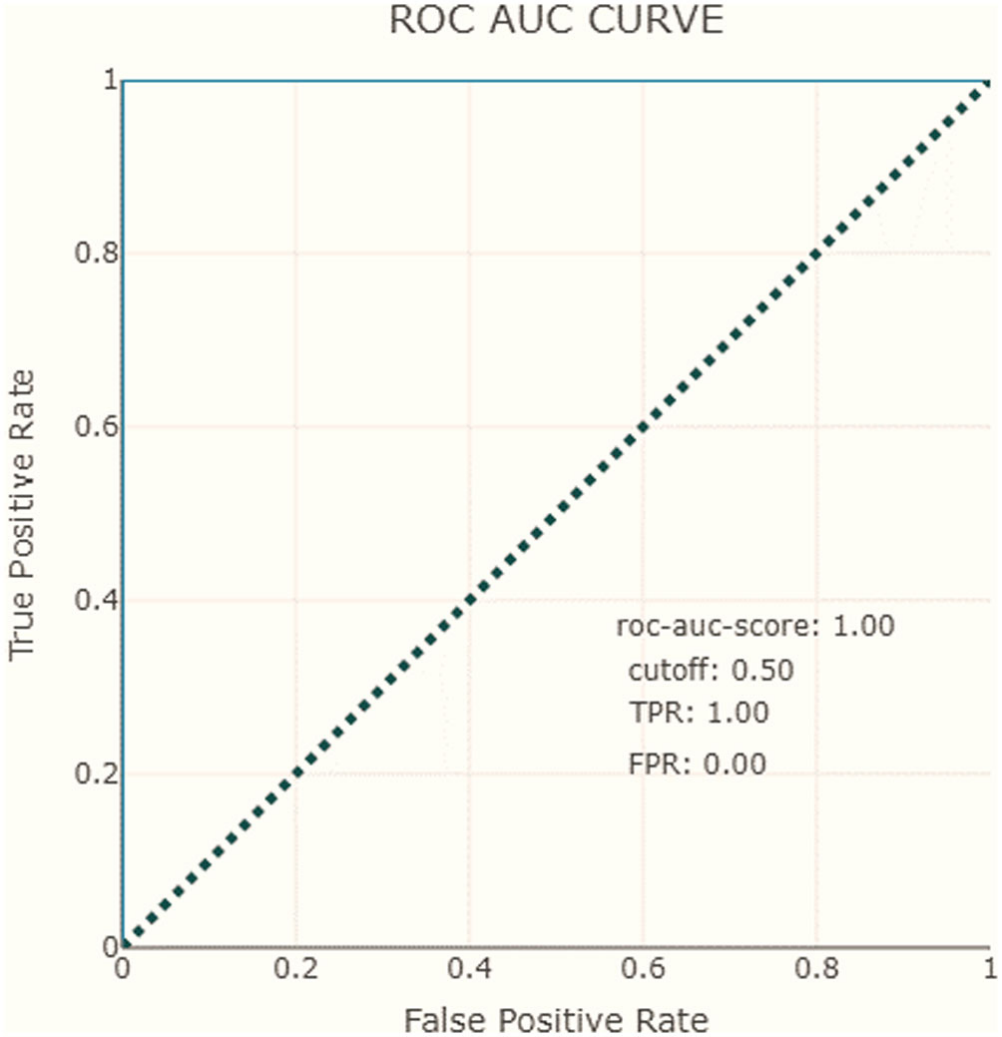
Receiver operating characteristics, area under the curve.

**Figure 9.**
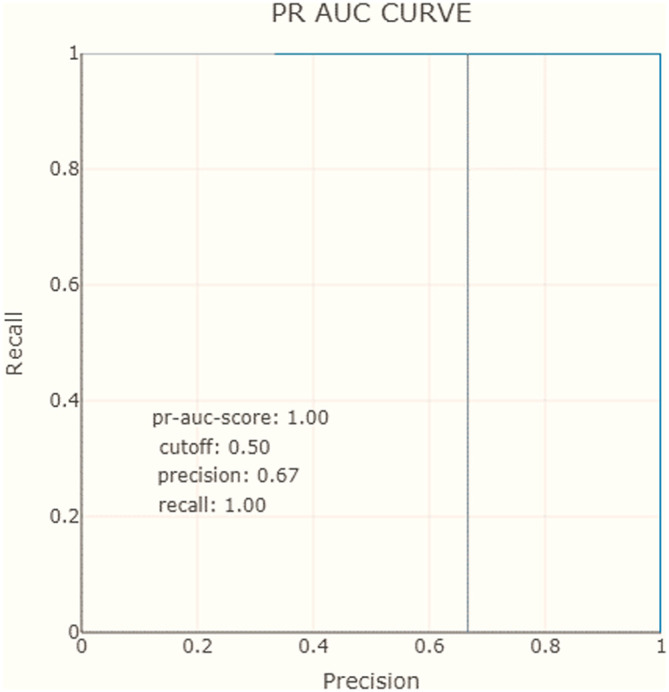
Precision-recall area under the curve.

Another evaluation measure considered in our dashboard is the AUC-ROC curve. For our model, the AUC score is equal to 1, which means that the classifier can correctly distinguish all points in the positive and negative classes (see Table 2). Similarly to ROC AUC, there are also the PR AUC, which combines PPV and TPR in a single visualization. This method explains at which recall our precision starts to fall fast, can help us to choose the threshold and deliver a better model. In Figures 8 and 9, we observe that for the negative class, the high precision and recall are maintained in almost the entire range of thresholds.

To recap, the XAI proposal includes precision and recall measures show that the model correctly predicts positive cases with an 80% precision and 100% recall. The AUC-ROC curve and PR AUC are also considered and show that the model can correctly distinguish all points in the positive and negative classes, with high precision and recall maintained throughout most of the range of thresholds.

**Figure 10.**
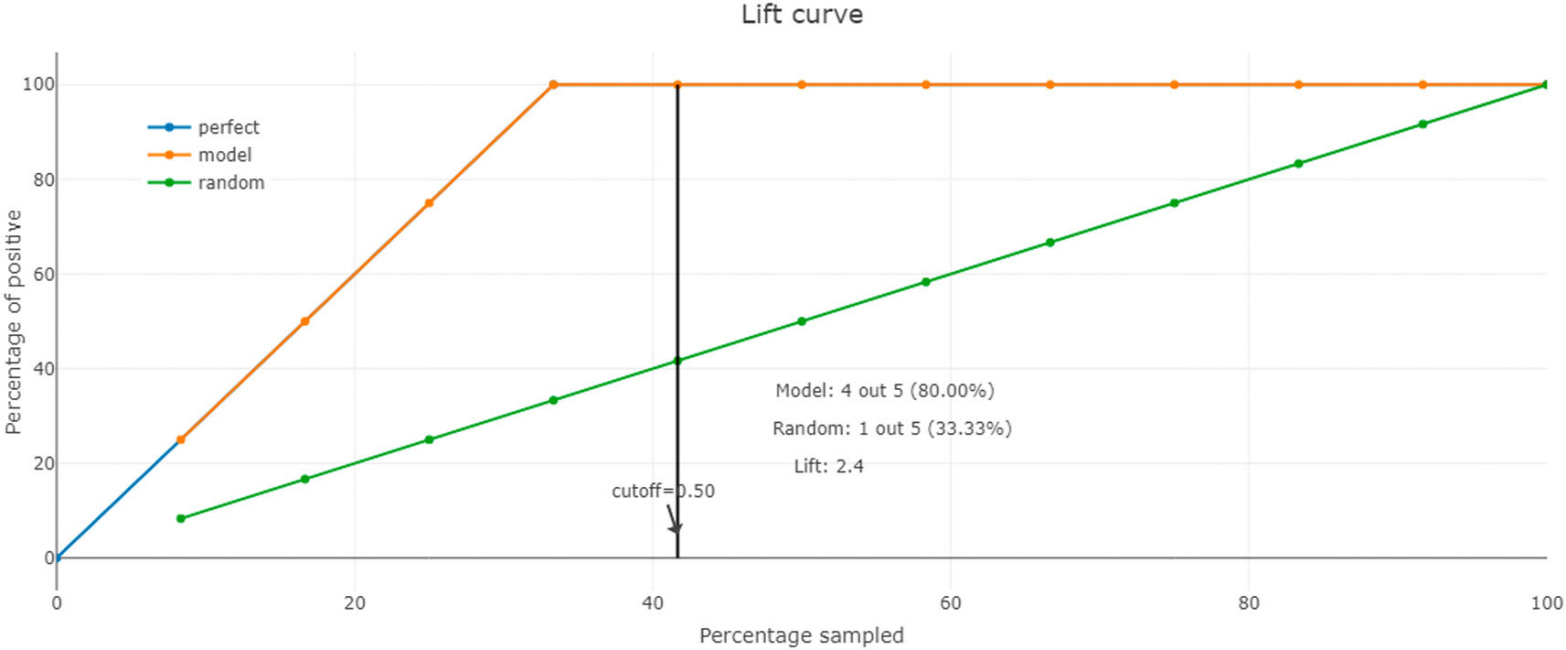
The lift curve.

In addition, we evaluate our model using the lift curve to compare its performance to that of a random model. The elevation curve shows the percentage of positive classes when selecting only observations with scores above the threshold compared to random selection of observations. In Figure 10, we see that our model’s performance is best from 30% of the population, which we can define as the threshold for optimal classification.

**Figure 11.**
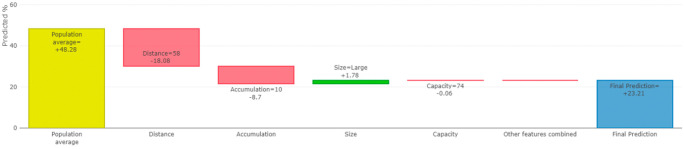
The feature contribution to our prediction.

**Figure 12.**
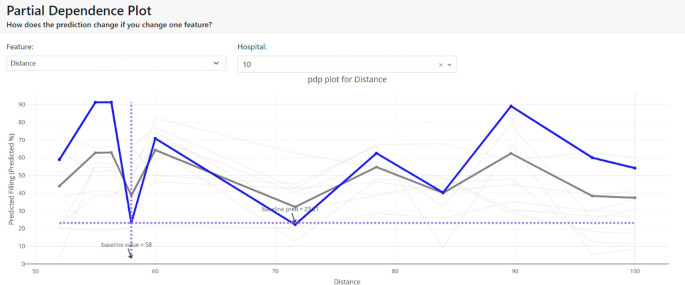
Partial dependence plot.

Furthermore, our XAI model can also shows the contribution of each individual feature to the prediction of bin fill. This allows us to explain exactly how each individual prediction was constructed from all the individual ingredients of the model for each hospital. For example, for node 10, the distance from the hospital to the warehouse, the accumulation rate, and the size of the hospital are the features that have the greatest effect on the prediction of bin filling (see Figure 11). We can also show how the model’s prediction would change if a particular feature were changed. Figure 12 shows the average effect (gray plot) and the effect of changing the feature for a single hospital (blue plot).

In conclusion, our XAI proposal addresses the need for transparency and interpretability in AI models. We have explained the importance of XAI in gaining end-user confidence by providing reliable and unbiased decision results. Our focus has been on the implementation of XAI in the prediction of medical waste bin fill levels.

By utilizing the XAI library and following a three-step process of data analysis, model evaluation, and production monitoring, we have developed an interpretable and user-friendly solution. Our interactive dashboard allows stakeholders to understand the inner workings of the machine learning models and provides various insights.

The feature importance analysis helps identify the relevant features for the model, such as distance, hospital size, vehicle capacity, and accumulation rate. The confusion matrix provides an overview of the model’s performance, while precision and recall measures demonstrate its ability to predict positive cases accurately, particularly in hazardous waste management where false alerts should be minimized. The AUC-ROC curve confirms the model’s ability to distinguish between positive and negative classes, while the lift curve compares its performance to a random model.

Furthermore, our XAI model allows for a granular analysis of predictions for each hospital, showcasing the contribution of each feature and demonstrating how changing a feature affects the prediction of bin fill. This comprehensive approach provides stakeholders with a clear understanding of the model’s decision-making process.

In summary, our XAI proposal not only ensures transparency and interpretability but also delivers reliable predictions for medical waste bin fill levels. By incorporating various evaluation measures and providing insightful visualizations, our solution empowers stakeholders to make informed decisions and optimize waste management practices.

### TDVRPTW for MWM

The medical waste recycling problem can be viewed as a general situation of the vehicle routing problem with time windows (VRPTW), which has been shown to be nondeterministic polynomial-time hard (NP-hard). ^68^

In this context, ^69^ proposed a particle swarm optimization (PSO) algorithm to optimize the biomedical waste collection problem in Coimbatore while minimizing the total collection time.

Similarly ^70^ also proposed a model with two phases to solve the medical waste collection problem with time windows. indeed, the first sub-model aims to find the best route for waste collection, and the second aims to allocate the resources of separation facilities to a set of recovery plants or landfill. The objective of this model is to minimize the total cost of transportation and maximize the revenue from recycling.

In the literature, there are also studies addressing the problems of routing vehicles while preventing and reducing the risks associated with MWM. For example, in Ref. 71 the authors proposed a model for the collection of several products while minimizing the risk of exposure to hazardous materials and the transportation costs affected by the route. In addition, the paper ^72^ considers the problem of routing multi-cycle medical waste recycling vehicles with time windows to prevent and reduce the risks associated with medical waste transportation. Furthermore, the authors of Ref. 73 proposed a bi-objective mathematical model addressing the problem of infectious and non-infectious medical waste transportation by considering the factor of stochastic contamination emissions during infectious waste transportation.

The literature review showed that a limited number of studies use vehicle routing models to solve the MWM problem, and that there is also a lack of consideration of the fact that medical waste must be fully treated within 48 hours.

In this context, our proposed model addresses the challenges of MWM by optimizing the collection routes (first sub model) and minimizing the transportation time to the treatment centers (second sub model). It accounts for the time-sensitive nature of medical waste treatment, as medical waste must be fully treated within 48 hours. It also considers the capacity of the collection vehicles and the treatment centers, and the regulations and policies related to MWM. In summary, this article presents a smart model for MWM that enhances the efficiency of waste collection and transportation while ensuring compliance with regulations and policies, and timely treatment of medical waste.

The smart routing problem for the collection of medical waste is based on the use of real-time data regarding the filling level of waste bins in hospitals, to define dynamic routes. This problem can be defined as follows: given a set of m hospitals with n waste bins, a set of v homogeneous vehicles, and a depot (where all vehicles start and end their routes), with distances $d_{\mathrm{ij}}$ for each edge $\left(i,j\right)$ . Each bin i has a maximum capacity $C_{i}$ .

**Figure 13.**
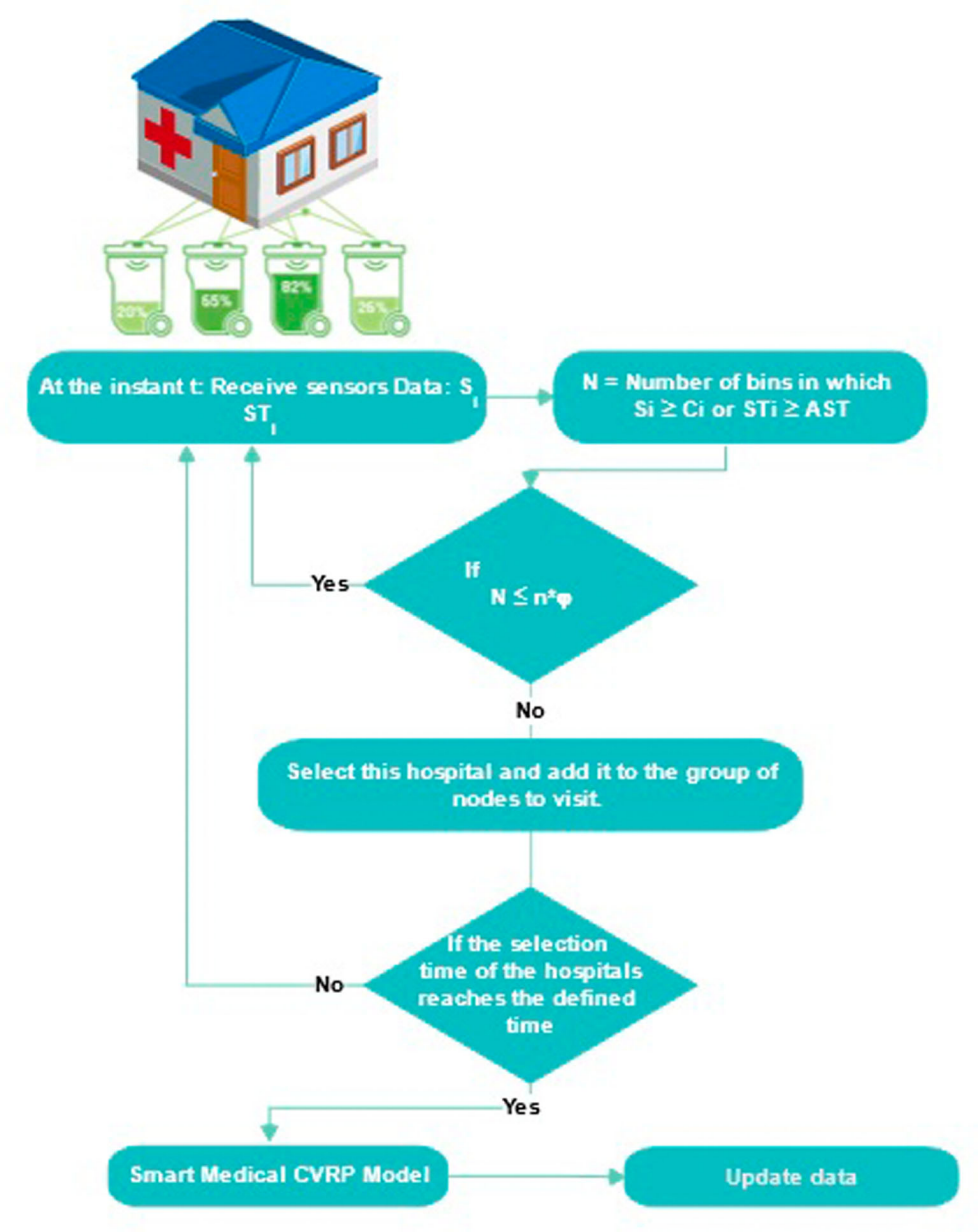
Smart collection approach.

To tackle this problem, we follow a three-step approach that involves first clustering hospitals, selecting those to be visited within a predetermined time frame, and finally applying a TDVRPTW model for the collection of medical waste. Indeed, in each sub-zone, we collect data from waste bins, then we calculate the number of bins that have exceeded their maximum capacity or the acceptable storage time of waste AST . If this number exceeds the desired service level ( φ , the tolerance for exceeding storage limits, depending on the type of waste), this hospital must be selected and added to the group of nodes to be visited. Then, we repeat this operation for each hospital for a predefined duration. This duration is calculated based on the fill level of the waste bins of each hospital, the acceptable storage time, and the travel time of the vehicles (in our case study, estimated at 4 hours). If the duration of hospital selection reaches the defined value, the TDVRPTW model for the collection of medical waste must be applied to the selected group of nodes (see Figure 13 & Algorithm 1).

Algorithm 1. **Notification algorithm for medical waste collection.**

**Input:** List of hospitals, Maximum capacity of waste bins $C_{i}$ , Acceptable waste storage time AST , Desired service level φ, Predefined duration of operation $t_{\max}$

**Output**: Group of nodes to be visited

Initialize the group of nodes to be visited to empty.

**while** ( **t <** $t_{\max}$ ) // (t time of the loop execution)

Calculate the number of waste bins that have exceeded their AST.

  
**If** this number exceeds the desired service level (φ), **do {**

  add this hospital to the group of nodes to be visited

  
**} end if**

**end**

**Return** the group of nodes to be visited gr

Apply the TDVRPTW model for the collection of medical waste of gr

**Figure 14.**
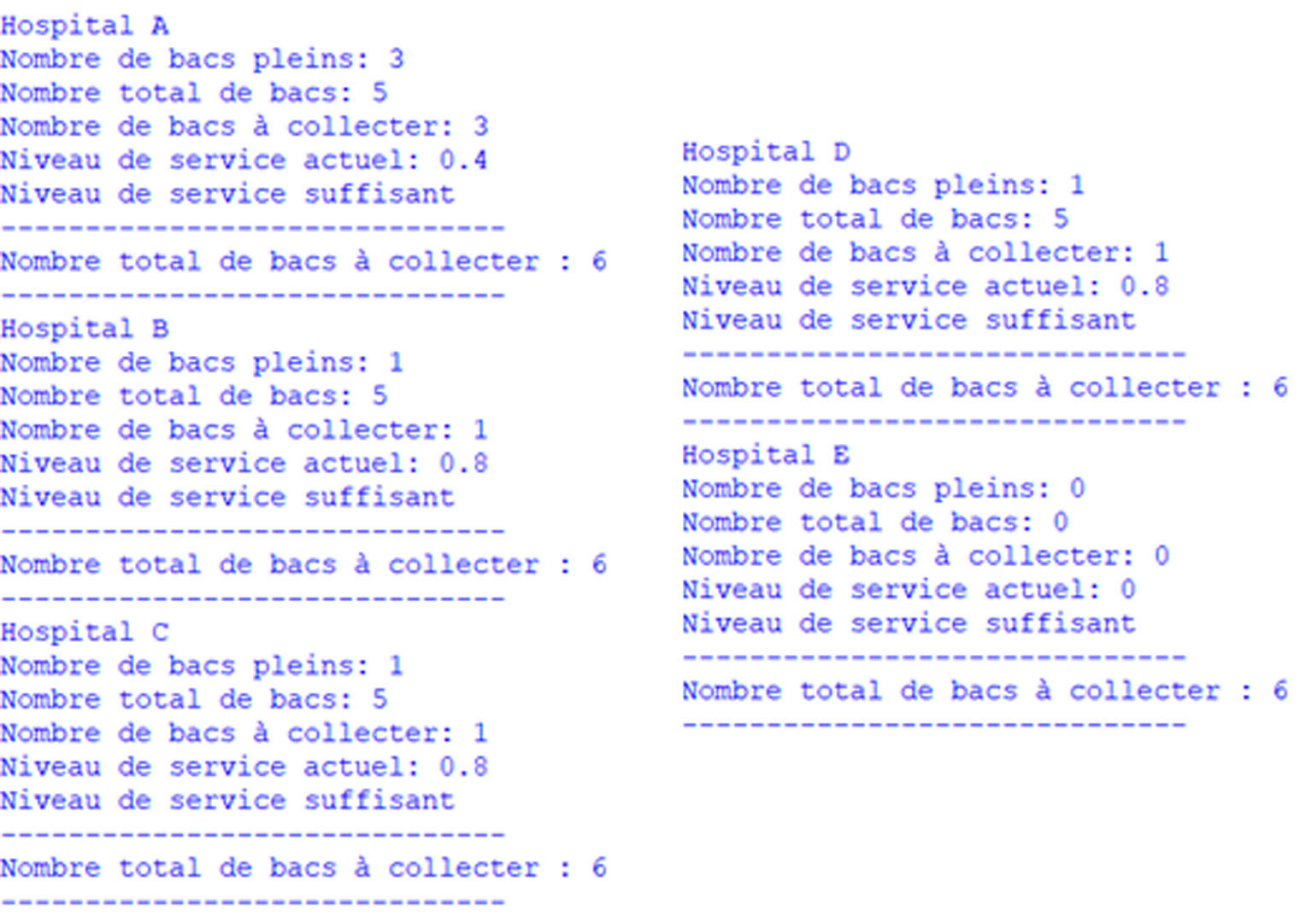
Algorithm 1 Result.

The outcomes of our algorithm are illustrated in Figure 14. The current level of service for each hospital is displayed, which indicates the number of non-full bins as a proportion of the total number of bins. If the current level of service falls below the desired level of service within the tolerance, an alert is issued to apply the TDVRPTW model to the selected nodes. Based on the results of our algorithm, the first route requires the collection of six waste bins.

To collect the bins designated by Algorithm 1, we will apply a TDVRPTW model that we will discuss in the next section. This model will optimize the collection of medical waste while taking into account the constraints of time windows, vehicle capacity, and travel time between nodes. By applying this model to the selected nodes, we can ensure that the collection process is efficient and timely. The results obtained by our algorithm will enable hospitals to maintain a high level of service while minimizing the impact of medical waste on the environment.

*First sub-module: The collection of medical waste*

**Figure 15.**
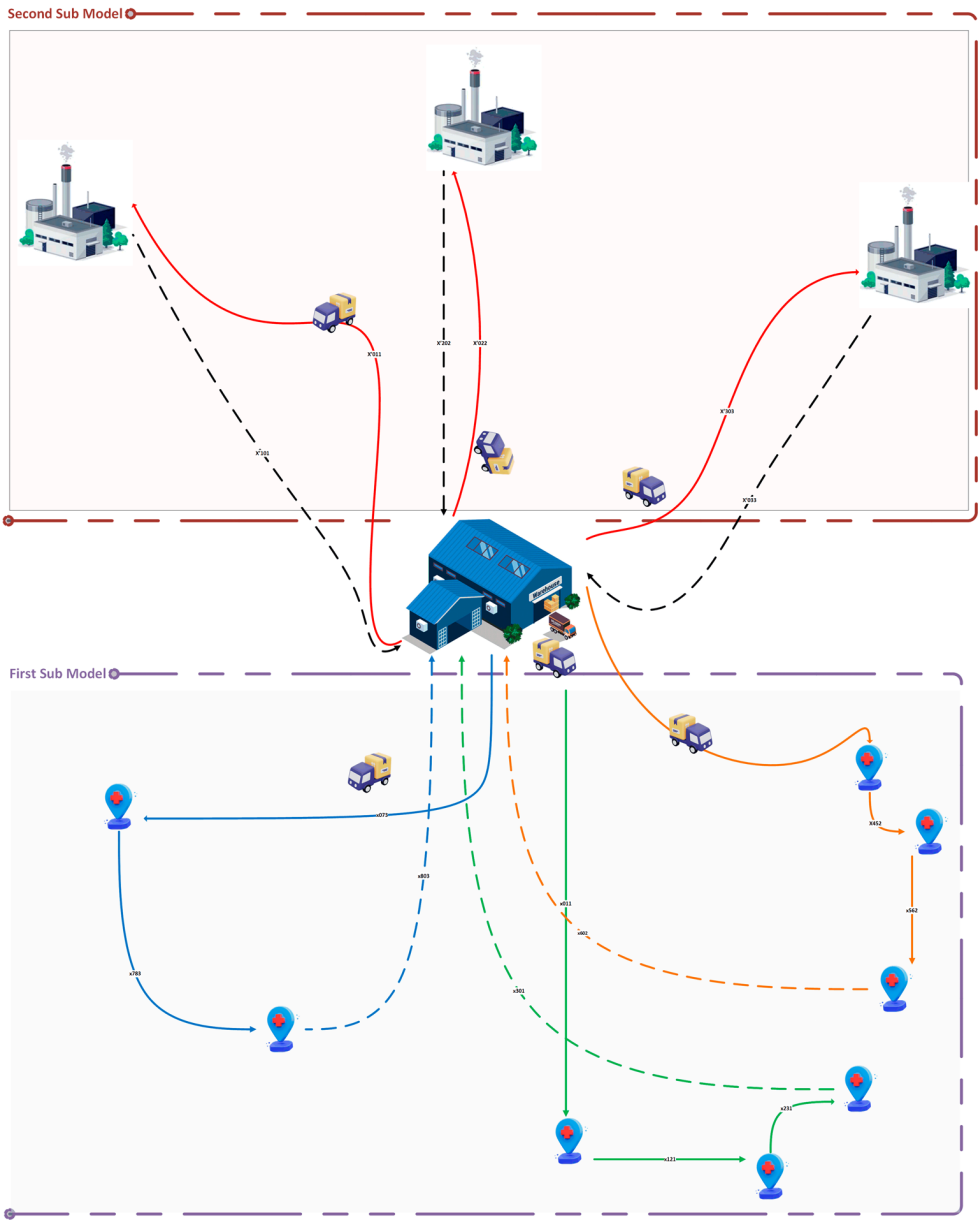
Two-commodity flow formulation representation.

In this section, the mathematical model of the first sub model is discussed in detail. Let $G=\left(V,A\right)\;$ be a graph where $A=\left\{\left(v_{i},v_{j}\right):i\neq j\right\}\;$ is an arc set and the vertex (node) set is $V=\left(v_{0},v_{1},\ldots ,v_{n+1}\right)$ , $v_{0}$ and $v_{n+1}$ denote the warehouse (see Figure 15). Each hospital has a quantity of waste to be collected and a time window within which the visit can be made. Vehicles have a limited capacity and must leave the depot to collect waste while minimizing the total distance travelled and respecting the time windows of each node.

To simplify the waste collection process, the assumptions made for this first sub-model are the following:

• The amount of waste to be collected for each hospital is known in advance through the proposed intelligent solution and cannot be split between several vehicles.
• The time windows of each node must be respected.
• The vehicles have a maximum capacity.
• The capacity of the warehouse is not limited (since it must be sized according to the sum of the average production of all the hospitals involved).

The notations used for this TDVRPTW sub module for medical waste collection are as follows:

**Index sets** $I=\left\{0,1,2,\ldots ,n\right\}$ set of n hospital and the real depot 0

Parameters

K number of available vehicles

$C_{\mathrm{ak}}$ vehicle capacity (in kg)

$d_{\mathrm{ij}}$ distance between node i and node j (in km)

${\mathrm{SH}}_{i}$ amount of waste in kg at Hospital i

$Q_{a}$ amount of collected waste in kg

${\mathrm{pop}}_{\mathrm{ij}}$ number of people in the bandwidth for waste along link *ij*

${\mathrm{RE}}_{\mathrm{jk}}$ Amount of waste in vehicle k at reaching j

${\mathrm{GE}}_{\mathrm{ij}}$ released gas rate between *i* and *j*

$f_{k}$ Vehicle fixed cost

β Travelling cost

$t_{\mathrm{wj}}$ waiting time at node *j*

$T_{\mathrm{ik}}$ the time when the vehicle *k* starts to serve node *j*

$T_{\mathrm{ak}}$ the arrival time of vehicle *k* at the warehouse

$T_{\mathrm{dk}}$ the departure time of vehicle *k* from the warehouse

$S_{i}$ service time in node *i*; (= 0; for *i* = 0)

$b_{i}$ start of the time window at node *i*

$e_{i}$ end of the time window at node *i*

$t_{\mathrm{ijp}}$ the traveled time between *ij* at period *p*

RI Risk exposure rate for transportation of waste

Decision variables

$x_{\mathrm{ijk}}$ binary variable indicating if edge $\left(i,j\right)$ is visited by vehicle k , $\left(i,j\;\epsilon \;I\right)$

$y_{k}$ binary variable indicating if the vehicle *k* is used

$Z_{\mathrm{jk}}$ binary variable indicating if node *j* is served by vehicle *k*

Objective function

$$Z=\beta \left(\sum_{i,\mathrm{j\epsilon}\;I}\sum_{k\;\epsilon \;K}d_{\mathrm{ij}}*x_{\mathrm{ijk}}\right)+\sum_{k\;\epsilon \;K}f_{k}*y_{k}$$ (5)

Constraints

$$\sum_{k\;\epsilon \;K}\sum_{i\;\epsilon \;I}x_{\mathrm{ijk}}=1\forall \mathrm{j\epsilon}\;I\;$$ (6)

$$\sum_{i\;\epsilon \;I}x_{\mathrm{ijk}}=Z_{\mathrm{jk}}\forall \mathrm{j\epsilon}\;I\;$$ (7)

$$\sum_{j\;\epsilon \;I}x_{\mathrm{ijk}}=Z_{\mathrm{ik}}\forall \mathrm{j\epsilon}\;I\;$$ (8)

$$x_{\mathrm{iik}}=0\forall \mathrm{k\epsilon}\;K$$ (9)

$$\sum_{i\;\epsilon \;I}x_{\mathrm{ipk}}=\sum_{i\;\epsilon \;I}x_{\mathrm{pjk}}\forall \mathrm{p\epsilon}\;I\;$$ (10)

$$\sum_{\mathrm{i\varepsilon I}}\sum_{j\;\epsilon \;I}\sum_{k\;\mathrm{\epsilon K}}{\mathrm{SH}}_{i}*x_{\mathrm{ijk}}=Q_{a}\forall \mathrm{j\epsilon}\;I\;$$ (11)

$$\sum_{i\;\epsilon \;I}\sum_{j\;\epsilon \;I}{\mathrm{SH}}_{i}*x_{\mathrm{ijk}}\leq {\mathrm{Ca}}_{k}*y_{k};\forall \mathrm{k\epsilon}\;K\;$$ (12)

$$\sum_{j\;\epsilon \;I}x_{0\mathrm{jk}}=y_{k}\forall k\;\epsilon \;K$$ (13)

$$\sum_{i\;\epsilon \;I}x_{i0k}=y_{k}\forall k\;\epsilon \;K$$ (14)

$$T_{\mathrm{ik}}+S_{i}+t_{\mathrm{ijp}}+t_{\mathrm{wj}}-T_{\mathrm{jk}}\leq \left(1-x_{\mathrm{ijk}}\right)*M\forall \mathrm{k\epsilon}\;K,\mathrm{i\epsilon I},\mathrm{j\epsilon I}$$ (15)

$$T_{\mathrm{ik}}+S_{i}+t_{i0p}-T_{\mathrm{ak}}\leq \left(1-x_{i0k}\right)*M\forall \mathrm{k\epsilon}\;K,\mathrm{i\epsilon I},\mathrm{j\epsilon I}$$ (16)

$$T_{\mathrm{dk}}+t_{0\mathrm{jp}}-T_{\mathrm{jk}}\leq \left(1-x_{0\mathrm{jk}}\right)*M\forall \mathrm{k\epsilon}\;K,\mathrm{i\epsilon I},\mathrm{j\epsilon I}$$ (17)

$$b_{i}*y_{k}\leq T_{\mathrm{ik}}\leq e_{i}*y_{k}\forall \mathrm{k\epsilon}\;K,\mathrm{i\epsilon I}$$ (18)

$$b_{0}*y_{k}\leq T_{\mathrm{ak}}\leq e_{0}*y_{k}\forall \mathrm{k\epsilon}\;K,\mathrm{i\epsilon I}$$ (19)

$$b_{0}*y_{k}\leq T_{\mathrm{dk}}\leq e_{0}*y_{k}\forall \mathrm{k\epsilon}\;K,\mathrm{i\epsilon I}$$ (20)

$$\sum_{k\;\epsilon \;K}y_{k}\leq K\;$$ (21)

$$x_{\mathrm{ij}}\;\epsilon \;\left\{0,1\right\}\forall i,j\in I,i\neq j$$ (22)

$$y_{\mathrm{ij}}\epsilon \;{\mathbb{R}}^{+}\forall i,j\in I,i\neq j$$ (23)

$$\mathrm{K\epsilon}\;{\mathbb{N}}^{*}$$ (24)

The objective of the TDVRPTW is represented by the objective function (5) which seeks to minimize the traveling cost. The constraints are defined as follows: (6), (7) and (8) all selected hospitals must be visited and they are visited only once; (9) serves to eliminate loops or isolated sub-tours; (10) a vehicle that arrives at a node should also depart from that node; (11) if a vehicle visits a hospital, all available waste must be collected; (12) the total demand in a particular route should not exceed the vehicle capacity; (13) and (14) indicate that vehicles should be taken out of the warehouse and returned to the warehouse at the end of the trip; (15), (16) and (17) regulate the start time of the service; (18), (19) and (20) vehicles should respect the time windows of the hospitals and the warehouse; (21) the number of vehicles should be less than or equal to the number available; the types of variables are indicated in constraint (22), (23) and (24).

The time-dependent vehicle routing problem with hospital medical waste pickup at different locations and time windows can be difficult to solve in real time, as the speed of the vehicles is not constant. To minimize the distance traveled, it is important to find efficient alternative routes using metaheuristics. In this study, a GA ^62^ was used to solve this problem, considering several vehicles with different capacities and varying travel times between different nodes. The performance of the GA used is compared with another existing algorithm for TDVRPTW developed by. ^63^

To evaluate the performance of the algorithm used, we conducted simulations using different sets of parameters (see Table 3). One of the simulation scenarios was carried out with mng = 1000 and pop = 100 to simulate a TDVRPTW model with 100 nodes and 25 vehicles. The score obtained was 559.6136551185149, indicating a high level of efficiency in the model. The distance traveled by the vehicles was 191.23298176125226, and the total travel time was 368.38067335726265. The runtime for the simulation was 84.48301577568054 seconds, which was reasonable considering the complexity of the problem. The number of trucks used was 2 out of 25, indicating that the model was able to optimize the routes efficiently. The routes taken by the vehicles were (5, 2, 7, 6, 8, 3, 1, 4) and (9, 10).

**Table 3.** Analysis of variance (ANOVA).

	(DL) Degrees of Freedom	Sum Car adjusted	Correction for the mean (CM) adjusted	F Value	P Value
Replication of Class	5	1313445	262689	38.75	0.000
Algorithm	1	697454	697454	102.89	0.000
Error	41	277937	6779		
Lack of fit	5	106082	21216	4.44	0.003
Pure error	36	171855	4774		
Total	47	2288836			

The GA used has shown its ability to generate good results despite the variations in key factors such as cluster structures, random aspects, and short or long scheduling horizons. The results obtained in our study have confirmed the effectiveness and robustness of our approach in tackling these complex challenges. By considering the different characteristics of the Solomon instances, the algorithm used has successfully found high-quality solutions, demonstrating its adaptability to diverse conditions and its efficient resolution of vehicle routing problems with time windows (see Table 3). These findings highlight the algorithm’s capacity to handle instance diversity and underscore its potential for real-world applications, where time constraints and operational conditions often change.

Furthermore, to justify our choice of algorithm, we performed a comparison of algorithms in terms of travelling cost. We used an ANOVA test followed by Tukey’s test to evaluate the significance of the difference in travelling cost between the different algorithms.

**Figure 16.**
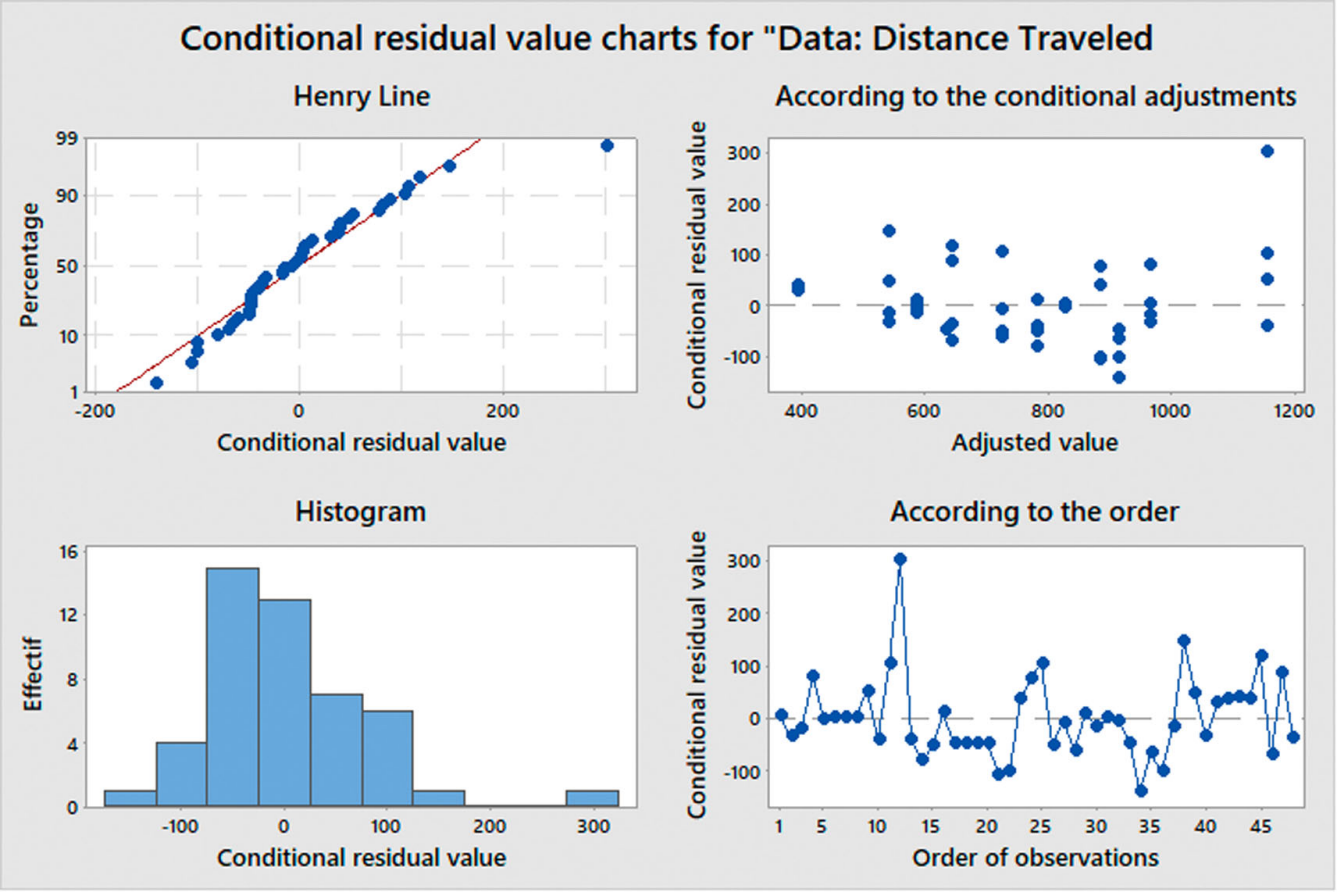
Conditional residual value charts for "data: distance traveled".

The ANOVA test is based on three main assumptions:

• Normality: The distribution of the errors should be normal (conditional residual value charts are used to check the assumptions of normality and homoscedasticity in the ANOVA model: see Figure 16).
• Homogeneity of variance: The variance of the errors should be equal across all groups.
• Independence: The observations should be independent of each other.

**Figure 17.**
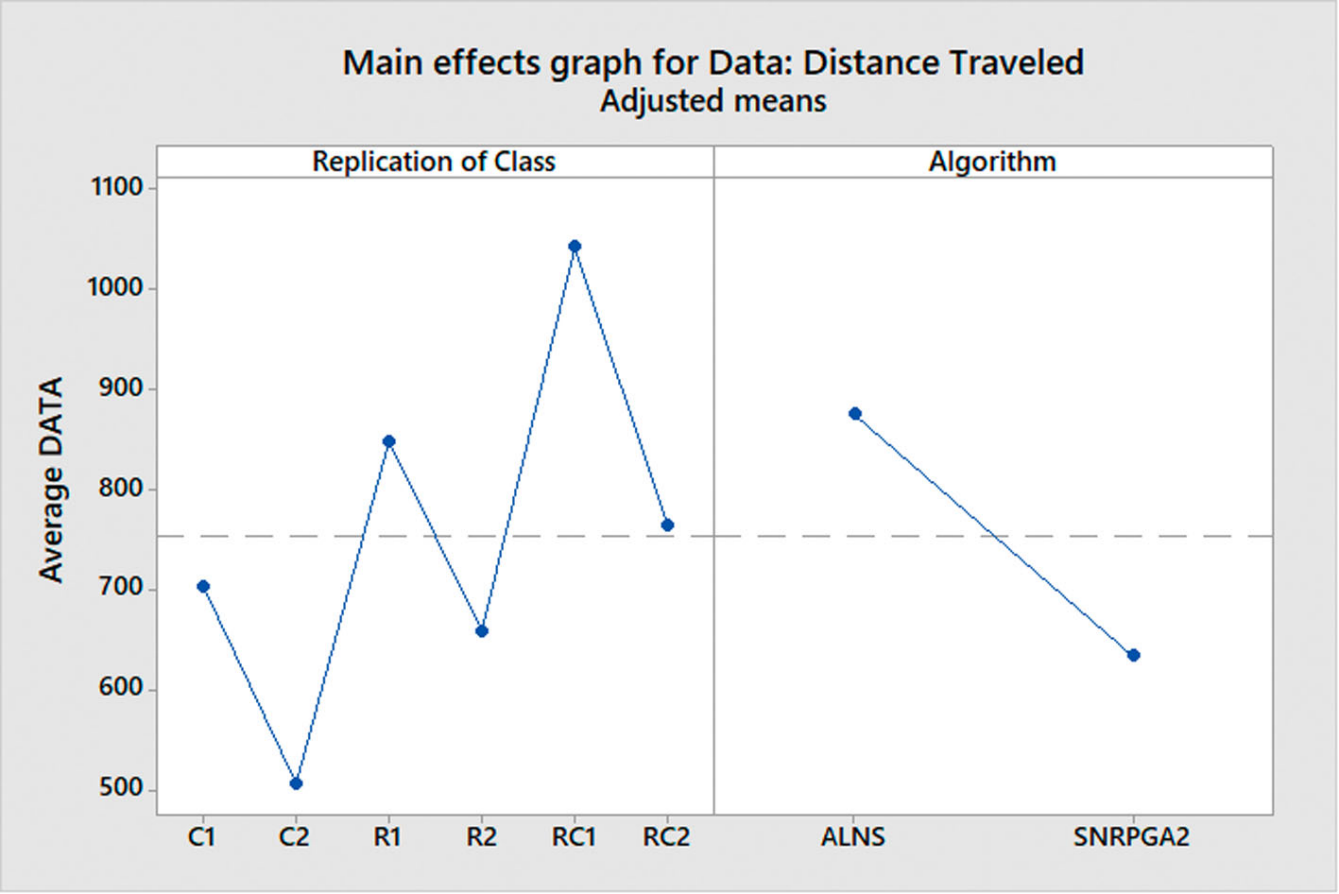
Main effects graph for data: distance traveled (adjusted means).

The ANOVA results show that there are significant differences between the means of the groups (see Tables 4 and 5). The main effect of the algorithm factor was found to be statistically significant (F = 102.89, p < 0.001), indicating that there are significant differences in the mean distances traveled between the two algorithms. The main effect of the replication of class factor was also found to be significant (F = 38.75, p < 0.001), suggesting that there are significant differences in the mean distances traveled across the six different classes (see Figure 17).

**Table 4.** Coefficients (ANOVA).

Term	Coeff	Coeff Error Term	T Value	P Value	FIV
Constant	754.0	11.9	63.45	0.000	
Replication of Class					
C1	-50.7	26.6	-1.91	0.064	*
C2	-247.0	26.6	-9.30	0.000	*
R1	94.2	26.6	3.55	0.001	*
R2	-94.7	26.6	-3.56	0.001	*
RC1	287.5	26.6	10.82	0.000	*
Algorithm					
ALNS	120.5	11.9	10.14	0.000	1.00

**Table 5.** Result - Simulation of TDVRPTW Model.

	Best solution for {ts_prob: 0.9, x_prob: 0.7, m_prob: 0.3, w_t: 1.0, mng: 100, pop_size: 100, init_method: random_sample, cache_gran: 1.0}					Best solution for {ts_prob: 0.9, x_prob: 0.7, m_prob: 0.3, w_t: 1.0, mng: 1000, pop_size: 100, init_method: random_sample, cache_gran: 1.0}				
	Score	Distance	Travel Time	Runtime (Sec)	Vehicles	Score	Distance	Travel Time	Runtime (Sec)	Vehicles
C101	27383	4022	23361	119	30	22004	3596	18408	11036	25
C102	23661	3937	19725	138	30	19894	3358	16536	10859	24
C103	21192	3756	17437	136	21	18829	3302	15527	10832	18
C104	17817	3654	14163	138	16	16347	2767	13580	11007	15
C105	25406	3830	21576	139	31	20580	3447	17132	10939	23
C106	24921	4157	20764	141	30	20541	3618	16922	10965	22
C107	22488	4237	18251	139	26	19325	3389	15936	10988	21
C108	22100	4138	17962	135	24	17526	3013	14513	11666	18
C109	20039	3962	16077	133	21	15873	2938	12934	12036	16
C201	56250	4493	51757	137	25	38374	3330	35044	11232	15
C202	44975	4198	40777	137	18	30976	3472	27504	11230	12
C203	44975	4493	51757	137	25	21847	3238	18609	11074	8
C204	20720	3673	17048	134	6	17483	3243	14240	11222	6
C205	52191	4054	48138	135	20	27807	3544	24262	11182	10
C206	42527	4345	38182	134	18	25854	3189	22666	12751	10
C207	41491	3819	37672	133	19	41491	3819	37672	133	19
C208	39911	3695	36215	138	15	23059	3470	19589	11381	8
R101	10003	3270	6733	138	42	8070	2672	5398	11254	33
R102	9164	3164	6001	120	37	7421	2561	4860	11133	27
R103	7944	2834	5110	110	29	6693	2432	4261	11189	25
R104	7349	2765	4583	110	24	5767	2180	3588	11157	18
R105	8748	3167	5582	113	35	6717	2367	4351	11230	27
R106	7977	2909	5068	109	30	6546	2362	4184	11918	25
R107	7667	2904	4764	109	27	6285	2360	3925	6342	21
R108	7045	2830	4214	108	21	5836	2243	3593	6240	19
R109	7769	3032	4736	108	29	6292	2431	3861	6203	24
R110	7549	2996	4553	147	29	6255	2411	3845	6256	24
R111	7410	2850	4560	165	25	5997	2243	3754	6254	21
R112	6564	2660	3904	132	23	5633	2204	3429	6268	20
R201	16256	3459	12797	78761	21	10834	2878	7956	6330	11
R202	13653	3298	10355	128	16	9383	2814	6569	6268	10
R203	10269	2901	7368	110	12	6914	2466	4448	6226	7
R204	7921	2944	4976	103	8	5725	2088	3637	6336	5
R205	11325	3158	8167	103	13	8364	2740	5623	6289	10
R206	10093	3113	6981	110	10	6887	2368	4519	9394	7
R207	8382	2870	5512	127	9	5529	1979	3550	12616	6
R208	6691	2629	4061	153	6	5132	1962	3170	11824	5
R209	10454	3334	7120	136	12	6654	2148	4507	11785	7
R210	10845	2932	7913	137	12	7080	2236	4844	11965	7
R211	7583	2635	4948	136	8	6161	2035	4126	11875	7
RC101	10626	4050	6576	141	40	8158	3040	5118	11966	30
RC102	10214	4012	6203	124	36	7356	2668	4688	12044	26
RC103	9261	3665	5596	115	30	6670	2618	4052	11953	22
RC104	8538	3521	5017	122	27	6391	2514	3877	11817	20
RC105	10378	3899	6479	120	38	7417	2732	4685	11891	27
RC106	9568	3873	5695	115	34	7052	2740	4312	11923	25
RC107	8541	3420	5121	118	30	6983	2798	4185	12380	25
RC108	8215	3350	4865	114	27	6477	2514	3963	12457	23
RC201	17233	4375	12858	116	22	11690	3353	8337	12344	13
RC202	14218	4089	10129	118	16	10191	3176	7015	12187	11
RC203	12183	4223	7960	118	13	8305	2667	5638	12165	9
RC204	9419	3528	5891	124	8	6884	2562	4321	12080	6
RC205	15690	4218	11472	113	18	10389	3291	7098	12225	10
RC206	13806	4211	9595	114	16	8383	2966	5418	12129	9
RC207	11392	3908	7484	105	12	7763	2548	5215	12273	9
RC208	9579	3403	6176	109	12	6887	2347	4540	14130	8

Additionally, the interaction effect between algorithm and replication of class was not significant (F = 0.25, p = 0.97), indicating that the effect of one factor does not depend on the level of the other factor. The lack of significant interaction effect implies that the main effects of Algorithm and Replication of Class can be interpreted independently.

In summary, the collective outcomes strongly indicate that the distance traveled is significantly influenced by two key factors: algorithm and replication of class. Consequently, when assessing algorithm performance, it is imperative to consider the impact of both these factors.

After conducting the ANOVA test, post-hoc tests can be performed to determine significant differences between groups. In this case, Tukey’s test was used to compare the means of the six classes for each algorithm (ALNS ^63^ or the algorithm used in this work “GA used” ^62^).

**Figure 18.**
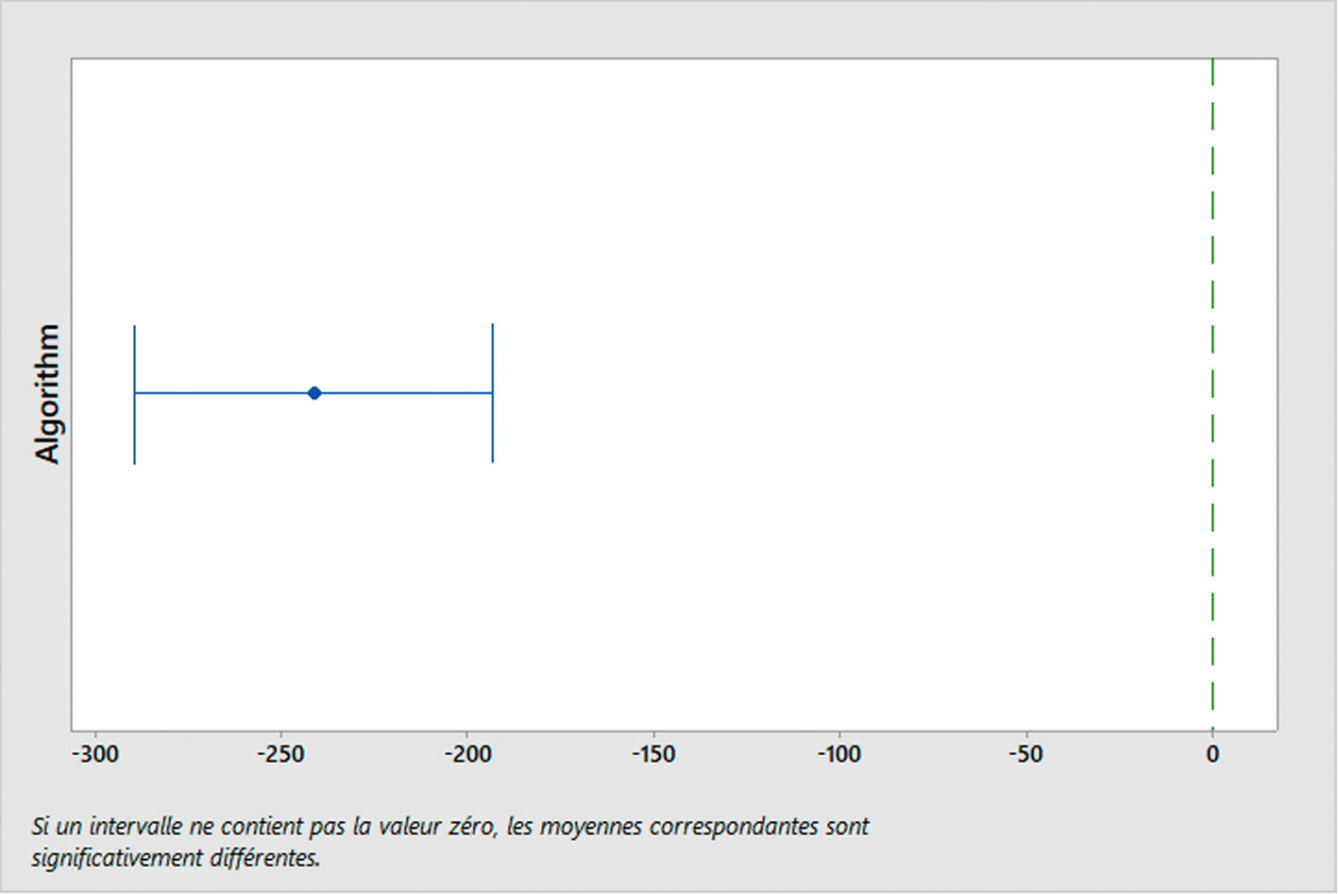
Concurrent Tukey 95% CIs.

The obtained results (see Table 6) reveal a noteworthy distinction in the average distances between the two algorithms (p < 0.001). Specifically, the “GA used” exhibits a significantly lower mean distance traveled compared to the ALNS algorithm (mean difference = -241.1, p < 0.001, 95% CI = -289.1 to -193.1) (refer to Figure 18). These findings strongly indicate that across all six problem classes, the “GA used” outperforms the ALNS algorithm in terms of distance traveled.

**Table 6.** Tukey simultaneity tests for differences between means.

Difference in levels algorithm	Difference of averages	Typical error of the difference	Simultaneous confidence interval 95%	T Value	Adjusted P Value
GA used - ALNS	-241.1	23.8	(-289.1; -193.1)	-10.14	0.000

To sum up, the outcomes of the ANOVA and Tukey tests reveal substantial disparities in the mean distances traveled across all six problem classes between the “GA used” and ALNS algorithms. The main effect of algorithm is statistically significant, indicating that the selection of algorithm significantly influences the distance traveled. Furthermore, the main effect of replication of class was also found to be significant, indicating that the problem class itself has a significant impact on the distance traveled.

The nonexistence of a substantial interaction effect between algorithm and replication of class indicates that the impact of these two factors on the distance traveled is separate and unrelated. These results suggest that both factors, algorithm and replication of class, should be considered when analyzing the performance of the algorithms.

Drawing upon these finding, we can infer that the “GA used” surpasses the ALNS algorithm in terms of the distance traveled across all six problem classes. This deduction is substantiated by the outcomes of the Tukey test, which demonstrate a significant decrease in the mean distance traveled by the “GA used” compared to the ALNS algorithm.

These results justify the choice of the “GA used” ^62^ over the widely used TDVRPTW algorithm in practical applications ^63^ where distance traveled is a critical performance metric. Future research could explore the performance of these algorithms on other metrics or in other problem domains.

*Second Sub-Module: Medical waste transportation to treatment centre*

The second sub-model can be framed as a TDVRPTW, in which the waste sorting center serves as the departure point for the transportation operations. Following waste sorting at the center, each vehicle is loaded with the waste stream that corresponds to its designated treatment destination.

The route planning process of each vehicle must account for the temporal and spatial constraints of waste transportation, as well as the loading capacity of the vehicles. Optimal route planning can minimize the overall costs of waste transportation while ensuring that each waste stream is transported to its respective treatment center for efficient and cost-effective treatment.

By leveraging TDVRPTW as a planning model, second sub-model can facilitate the optimization of waste transportation efficiency while simultaneously reducing environmental impacts (see Table 7). This serves to advance the development of a cleaner and more sustainable environment for the benefit of future generations.

**Table 7.** Comparative table of the proposed solution and the traditional solution (trip for transport of medical waste to treatment centers).

Indicator	Distribution without sorting	Cross-docking with sorting
Total treatment cost	100 000€	80 000€
Number of treatment centers	4	4
Initial investment cost	10 000€	50 000€
Transport cost	20 000€	15 000€
Total treatment time	40 hours	30 hours
Environmental cost	Risk of cross-contamination	Reduction of the risk of cross-contamination
Capacity utilization of each treatment center:	All centers are used to their full capacity	The capacity of each center is optimized through the distribution of waste by type

To provide further elucidation of the second sub-model, a detailed discussion of the mathematical model is presented. Let $G=\left(V,A\right)\;$ be a graph where $A=\left\{\left(v_{i},v_{j}\right):i\neq j\right\}\;$ is an arc set and the vertex (node) set is $V=\left(v_{0},v_{1},\ldots ,v_{n+1}\right)$ , $v_{0}$ and $v_{n+1}$ denote the warehouse (see Figure 15). In this waste distribution problem, every amount of waste must be transported to its corresponding treatment center $v_{i}$ , which has a designated time window for the visit to take place.

The assumptions made for this sub-model are the following:

• The time windows of each node must be respected.
• The vehicles have a maximum capacity.
• The capacity of the center treatment is not limited.

The notations used for this TDVRPTW sub module for medical waste distribution are as follows:

Index sets $I=\left\{0,1,2,\ldots ,n\right\}$ set of n center treatment and the real depot 0

Parameters

K number of available vehicles

$C\prime_{\mathrm{ak}}$ vehicle capacity (in kg)

$d\prime_{\mathrm{ij}}$ distance between node i and node j (in km)

${\mathrm{WT}}_{i}$ amount of waste in kg be transported to the treatment center i

$Q\prime_{a}$ amount of transported waste in kg

${\mathrm{EF}}_{\mathrm{ip}}$ Emission Factor: the rate at which pollutants p are released per unit of waste treated in center i

$\gamma_{p}$ Marginal Cost of Pollution: the cost associated with each unit of pollutant released, which takes into account the environmental and health impacts of the pollution.

${\mathrm{RE}}_{\mathrm{jk}}$ Amount of waste in vehicle k at reaching j

${\mathrm{GE}}_{\mathrm{ij}}$ released gas rate between *i* and *j*

$f_{k}$ fixed cost = Vehicles Usage Cost + Outsourcing Costs

β Travelling cost

$t_{\mathrm{wj}}$ waiting time at node *j*

$T_{\mathrm{ik}}$ the time when the vehicle *k* starts to serve node *j*

$T_{\mathrm{ak}}$ the arrival time of vehicle *k* at the warehouse

$T_{\mathrm{dk}}$ the departure time of vehicle *k* from the warehouse

$S_{i}$ service time in node *i*; (= 0; for *i* = 0)

$b_{i}$ start of the time window at node *i*

$e_{i}$ end of the time window at node *i*

$t_{\mathrm{ijp}}$ the traveled time between *ij* at period *p*

RI Risk exposure rate for transportation of waste

Decision variables

$x\prime_{\mathrm{ijk}}$ binary variable indicating if edge $\left(i,j\right)$ is visited by vehicle k , $\left(i,j\;\epsilon \;I\right)$

$y\prime_{k}$ binary variable indicating if the vehicle *k* is used

$Z\prime_{\mathrm{jk}}$ binary variable indicating if node *j* is visited by vehicle *k*

Objective function

$$Z=\beta \left(\sum_{i,\mathrm{j\epsilon}\;I}\sum_{k\;\epsilon \;K}d_{\mathrm{ij}}*x\prime_{\mathrm{ijk}}\right)+\sum_{k\;\epsilon \;K}f_{k}*y_{k}+\sum_{i\in I}{\mathrm{WT}}_{i}*\sum_{p\;\epsilon \;P}{\mathrm{EF}}_{\mathrm{ip}}*\gamma_{p}$$ (25)

Constraints

$$\sum_{i\;\epsilon \;I}x\prime_{\mathrm{ijk}}=Z\prime_{\mathrm{jk}}\forall \mathrm{j\epsilon}\;I\;$$ (26)

$$\sum_{j\;\epsilon \;I}x\prime_{\mathrm{ijk}}=Z\prime_{\mathrm{ik}}\forall \mathrm{j\epsilon}\;I\;$$ (27)

$$x\prime_{\mathrm{iik}}=0\forall \mathrm{k\epsilon}\;K$$ (28)

$$\sum_{i\;\epsilon \;I}x\prime_{\mathrm{ipk}}=\sum_{i\;\epsilon \;I}x\prime_{\mathrm{pjk}}\forall \mathrm{p\epsilon}\;I\;$$ (29)

$$\sum_{\mathrm{i\varepsilon I}}\sum_{j\;\epsilon \;I}\sum_{k\;\mathrm{\epsilon K}}{\mathrm{WT}}_{i}*x\prime_{\mathrm{ijk}}=Q\prime_{a}\forall \mathrm{j\epsilon}\;I\;$$ (30)

$$\sum_{i\;\epsilon \;I}\sum_{j\;\epsilon \;I}{\mathrm{WT}}_{i}*x\prime_{\mathrm{ijk}}\leq C\prime a_{k}*y_{k};\forall \mathrm{k\epsilon}\;K\;$$ (31)

$$\sum_{j\;\epsilon \;I}x\prime_{0\mathrm{jk}}=y\prime_{k}\forall k\;\epsilon \;K$$ (32)

$$\sum_{i\;\epsilon \;I}x\prime_{i0k}=y\prime_{k}\forall k\;\epsilon \;K$$ (33)

$$T_{\mathrm{ik}}+S_{i}+t_{\mathrm{ijp}}+t_{\mathrm{wj}}-T_{\mathrm{jk}}\leq \left(1-x\prime_{\mathrm{ijk}}\right)*M\forall \mathrm{k\epsilon}\;K,\mathrm{i\epsilon I},\mathrm{j\epsilon I}$$ (34)

$$T_{\mathrm{ik}}+S_{i}+t_{i0p}-T_{\mathrm{ak}}\leq \left(1-x\prime_{i0k}\right)*M\forall \mathrm{k\epsilon}\;K,\mathrm{i\epsilon I},\mathrm{j\epsilon I}$$ (35)

$$T_{\mathrm{dk}}+t_{0\mathrm{jp}}-T_{\mathrm{jk}}\leq \left(1-x\prime_{0\mathrm{jk}}\right)*M\forall \mathrm{k\epsilon}\;K,\mathrm{i\epsilon I},\mathrm{j\epsilon I}$$ (36)

$$b_{i}*y\prime_{k}\leq T_{\mathrm{ik}}\leq e_{i}*y\prime_{k}\forall \mathrm{k\epsilon}\;K,\mathrm{i\epsilon I}$$ (37)

$$b_{0}*y\prime_{k}\leq T_{\mathrm{ak}}\leq e_{0}*y\prime_{k}\forall \mathrm{k\epsilon}\;K,\mathrm{i\epsilon I}$$ (38)

$$b_{0}*y\prime_{k}\leq T_{\mathrm{dk}}\leq e_{0}*y\prime_{k}\forall \mathrm{k\epsilon}\;K,\mathrm{i\epsilon I}$$ (39)

$$\sum_{k\;\epsilon \;K}y\prime_{k}\leq K\;$$ (40)

$$x\prime_{\mathrm{ij}}\;\epsilon \;\left\{0,1\right\}\forall i,j\in I,i\neq j$$ (41)

$$y\prime_{\mathrm{ij}}\epsilon \;{\mathbb{R}}^{+}\forall i,j\in I,i\neq j$$ (42)

$$\mathrm{K\epsilon}\;{\mathbb{N}}^{*}$$ (43)

The objective function (25) of the TDVRPTW problem for waste transportation includes three main cost components: the travelling cost, the fixed cost, and the pollution cost of treatment centers. The travelling cost refers to the cost incurred for each unit of distance travelled by the vehicle, while the fixed cost includes the cost of using vehicles and the outsourcing costs. Finally, the pollution cost is a measure of the environmental impact of the waste treatment process, which takes into account the marginal cost of each unit of pollutant released by the treatment center. The optimization of the objective function can help to minimize the cost of waste transportation while ensuring that each waste is delivered to the appropriate treatment center for effective and efficient treatment, contributing to a cleaner and more sustainable environment.

The constraints used for this second sub model are similar to those previously mentioned for hospital waste collection. These constraints ensure that all treatment centers are visited exactly once by vehicle k (26- 27), prevent loops or isolated sub-tours (28), require that a vehicle that arrives at a center also departs from it (29), mandate the distribution of all available waste to the appropriate center (30), limit the total demand in a particular route to the vehicle capacity (31), dictate that vehicles start and end at the warehouse (32- 33), regulate the start time of service (33- 36), ensure that vehicles respect time windows of the centers and warehouse (37- 39), limit the number of vehicles to those available (40), and specify the types of variables used (41- 43).

To solve the TDVRPTW problem of waste distribution to treatment centers, we utilized the same GA described in the previous sections.

The fitness function for the GA was defined as follows:

$$Z=\beta \left(\sum_{i,\mathrm{j\epsilon}\;I}\sum_{k\;\epsilon \;K}d_{\mathrm{ij}}*x\prime_{\mathrm{ijk}}\right)+\sum_{k\;\epsilon \;K}f_{k}*y_{k}+\sum_{i\in I}{\mathrm{WT}}_{i}*\sum_{p\;\epsilon \;P}{\mathrm{EF}}_{\mathrm{ip}}*\gamma_{p}$$ (25)

The travel cost encompasses expenses related to fuel and vehicle maintenance, whereas the fixed vehicle cost comprises the costs associated with vehicle acquisition and upkeep. Outsourcing costs pertain to the expenses incurred when subcontracting certain aspects of the waste treatment process. Pollution costs encompass the financial implications of the environmental impact caused by the waste treatment process.

The GA was implemented in Python(RRID:SCR_008394) (v 3.9.13), and the optimization process involved several steps. First, an initial population of candidate solutions was randomly generated. Each solution represented a possible distribution of waste to treatment centers. Then, the fitness function was applied to each solution, and the solutions were ranked based on their fitness scores.

Next, a selection process was used to identify the fittest solutions, which were then used as the basis for creating new candidate solutions through a process of crossover and mutation. The crossover process involved combining elements of two parent solutions to create a new child solution. The mutation process involved randomly altering one or more elements of a solution to create a new variant.

This process was repeated for a set number of generations, with each generation producing a new population of candidate solutions. The algorithm terminated when the fitness score of the best solution in the population met a predefined threshold, or when a maximum number of generations was reached.

Overall, the GA approach proved to be effective in optimizing the distribution of waste to treatment centers while minimizing costs and environmental impact. The results obtained from the GA could be used to inform waste management policies and practices, and to promote more sustainable waste management practices for the benefit of future generations.

Our study case consists of 10 treatment centers, 4 available waste transport vehicles, each with a capacity of 250 units, this scenario is based on the context of Casablanca. After applying the GA with the constraints and fitness function described above, we obtain an optimal solution that minimizes the total cost of distributing medical waste. The first round consists of transporting the collected waste to the first treatment center, which specializes in the treatment of infectious waste. The other two vehicles begin their rounds at the second treatment center, which specializes in the treatment of radioactive waste. They collect radioactive waste from all nearby hospitals and deliver it to the second treatment center. The cumulative cost of this approach is determined by combining the expenses associated with vehicle travel, fixed vehicle costs, waste collection outsourcing, and the environmental impact resulting from vehicle operations and processing centers.

Implementing this optimal solution has resulted in a reduction in both waste transportation costs and the expenses linked to pollution emanating from vehicles and treatment centers (refer to Table 7).

### MWM through cross-docking

To illustrate the benefits of the warehouse for cross-docking, we used a simulation modeling using Simul8 (v 3.0). ^66^ The simulation aimed to replicate real-world MWM scenarios while rigorously testing the impact of various parameters (vehicle capacity, time windows, cross-docking center size, transport costs, processing times, initial investment costs, cross-contamination rates, etc). We ran the simulation using two scenarios for each waste management method, distribution without sorting and cross-docking with sorting.

In the first case, where waste is distributed without prior sorting, it is possible that some types of waste are sent to inappropriate processing centers, which can result in additional costs for processing these wastes. In addition, cross-contamination between different types of waste can lead to environmental and public health problems. In the second case, setting up a warehouse for cross-docking with waste sorting can reduce processing costs, better use resources, and reduce environmental impacts. Indeed, each type of waste can be sent to the specialized processing center for its specific treatment, which allows for better resource management and reduces cross-contamination. However, setting up a warehouse for cross-docking may require larger initial investments and more complex coordination between the different actors involved in the waste processing process.

In this example, distribution without sorting of medical waste resulted in a total processing cost of €100,000, whereas setting up a cross-docking warehouse with sorting reduced this cost to €80,000. However, the initial investment cost for setting up the cross-docking warehouse was €50,000, five times higher than the initial investment cost for distribution without sorting. Transport cost was also reduced from €20,000 to €15,000 thanks to the optimization of MWM by cross-docking with sorting. In terms of processing time, cross-docking with sorting allowed a significant reduction of 30 hours, compared to the 40 hours required for distribution without sorting. Finally, the risk of cross-contamination was reduced thanks to the setting up of a cross-docking warehouse with sorting, which allowed each type of waste to be sent to the appropriate processing center.

In the case of unsorted distribution of medical waste, waste of different types was sent to treatment centers that were not specialized in their specific treatment. This can result in additional costs for the treatment of these wastes, as treatment centers must adapt their equipment and personnel to handle these types of wastes that were not originally planned for. Additionally, cross-contamination between different types of wastes can lead to environmental and public health issues, thereby increasing costs to manage these problems. On the other hand, in the case of setting up a cross-docking warehouse with sorting, each type of waste is sent to the specialized treatment center for its specific treatment, allowing for better resource management and a reduction in cross-contamination. This can reduce overall treatment costs as treatment centers can specialize in one type of waste and optimize their equipment and personnel to specifically treat that type of waste. Furthermore, the reduction in cross-contamination can reduce costs associated with managing environmental and public health issues. As a result, the total cost of treatment was reduced by €20,000 with the implementation of a cross-docking warehouse with sorting, compared to unsorted distribution of medical waste.

Furthermore, with the implementation of a cross-docking warehouse with sorting, vehicles were used more efficiently. Waste was sorted based on its treatment type and destination, allowing for optimal filling of vehicles and a reduction in the number of required trips. This led to a reduction in transportation costs from €20,000 to €15,000.

Moreover, cross-contamination is a significant problem in MWM. It can lead to the spread of diseases and infections, as well as contamination of the environment. For example, if infectious wastes are mixed with non-infectious wastes, this can lead to disease spread. Similarly, if chemical wastes are mixed with organic wastes, this can lead to dangerous chemical reactions and the production of toxic gases. In fact, when chemical wastes are mixed with organic wastes, chemical reactions can occur, leading to the production of toxic gases. For instance, if wastes containing chlorine-containing chemicals are mixed with organic wastes such as food, a chemical reaction can occur to produce hydrogen chloride gas (HCl), which is toxic to humans and the environment. Another example is the reaction between wastes containing acidic chemicals and wastes containing alkaline chemicals, which can produce toxic gases such as ammonium chloride or hydrochloric acid gas. By using the cross-docking warehouse with waste sorting, each type of waste is sent to the appropriate treatment center, reducing the risk of cross-contamination and ensuring safer and more efficient management of medical waste.

On the other hand, although the initial investment for the warehouse solution is higher, optimizing the MWM process can lead to long-term savings by reducing treatment and transportation costs, as mentioned earlier. Additionally, using a cross-docking warehouse with waste sorting allows each type of waste to be sent to the specialized treatment center for its specific treatment, which improves the quality of treatment and can enable better recovery of raw materials. Not to mention that the reduction in the risk of cross-contamination through the separation of different types of wastes can have benefits in terms of public health and environmental safety.

In conclusion, when comparing the two approaches, it was found that the implementation of a cross-docking warehouse allowed for a reduction in medical waste treatment costs, better resource utilization, and a reduction in environmental impact compared to unsorted distribution. The results showed that the implementation of a cross-docking warehouse can be an effective solution for MWM in hospitals and healthcare centers.

## Conclusions

This article has thoroughly addressed the issue of MWM using vehicle routing models and cross-docking techniques. It has presented two sub-models, one for medical waste collection and the other for transportation to treatment centers, highlighting the importance of the cross-docking process for efficient coordination of these stages.

The article has emphasized the use of a smart cross-docking center to facilitate seamless transition of collected waste between collection and transportation vehicles without intermediate storage. Additionally, the author has proposed the specialization of treatment centers to focus on specific types of waste treatment, which would enhance the overall efficiency of the waste management system.

The evaluation of the algorithm’s performance demonstrated its ability to reduce the number of vehicles and distance traveled. The utilization of ANOVA and Tukey tests confirmed the statistical significance of these improvements, further supporting the effectiveness of the proposed approach.

The article also leveraged IoT technologies, including sensors and smart objects, to enable real-time monitoring of waste levels and optimize collection schedules.

Furthermore, the article incorporated XAI techniques to provide transparent and interpretable explanations for the decision-making process of the proposed models. This ensures that the stakeholders involved can understand and trust the recommendations made by the system.

In conclusion, this article presents an innovative approach to MWM by integrating vehicle routing models, cross-docking techniques, IoT utilization, and XAI. This approach enhances operational efficiency, reduces costs, and promotes better resource utilization. Future prospects could explore further optimization of route planning and the integration of advanced AI techniques for even more sophisticated MWM.

## Data Availability

We have a collection of synthetic datasets known as the “Solomon instances”, ^65^ which consist of 56 instances featuring 100 clients with time windows http://www.vrp-rep.org/references/item/solomon-1987.html. These instances are categorized into six sets based on their specific attributes: The specific datasets mentioned in this project are categorized into six sets based on their attributes:
• C1….txt: Clustered instances with a short scheduling horizon. • C2….txt: Clustered instances with a long scheduling horizon. • R1.….txt: Random instances with a short scheduling horizon. • R2….txt: Random instances with a long scheduling horizon. • RC1….txt: Random and clustered instances with a short scheduling horizon. • RC2….txt: Random and clustered instances with a long scheduling horizon. This dataset represents a standardized set of test instances for evaluating and comparing the algorithm for solving TDVRPTW. This dataset is characterized by the following key features:
• Customers and time windows: Each instance in the dataset represents a set of customers (depots) with specific demand quantities and time windows within which they can be serviced. • Vehicle capacity and number: The dataset also specifies the capacity of the vehicles used for deliveries and the number of available vehicles. • Coordinates: For each customer, the dataset includes its geographical coordinates (usually represented as (x, y) coordinates in a Euclidean space). • Service Duration: The time taken to service each customer is also provided. • Depot Information: The dataset contains information about the depot (depot coordinates, available vehicles, etc.). • Objective Value: For each instance, the optimal or best-known solution (i.e., the minimum route duration) is provided. This value is used to evaluate the quality of solutions obtained by different algorithms. Additionally, we have an extension of Solomon’s instances that incorporates time-dependence, as introduced by Ichoua *et al.* ^74^ Indeed, the travel times between locations (customers or depots) are not constant but vary depending on the time of day. This extension aims to make the vehicle routing optimization more realistic by considering the dynamic nature of travel times in real-world scenarios. This extension includes three different scenarios, each varying in the degree of time-dependence:
• t_dep.dat: Speed matrix for the first scenario, which exhibits the lowest level of time-dependence. • t_dep2.dat: Speed matrix for the second scenario. • t_dep3.dat: Speed matrix for the third scenario, characterized by the highest degree of time-dependence. Zenodo: Zineb-bdg/Medical-waste-management-system-in-a-smart-city-using-XAI-and-Vehicle-Routing-Optimization-Data-: Initial Implementation. https://doi.org/10.5281/zenodo.8157394 ^57^ This project contains the following underlying data:
• Waste_test.csv • Waste_train.csv Data are available under the terms of the Creative Commons Zero “No rights reserved” data waiver (CC0 1.0 Public domain dedication).

## Software availability

**Archived source code at the time of publication**: https://doi.org/10.5281/zenodo.8157394. ^57^

**Source code available from**: https://github.com/Zineb-bdg/Medical-waste-management-system-in-a-smart-city-using-XAI-and-Vehicle-Routing-Optimization-Data

**License:** This project is licensed under the Creative Commons Zero v1.0 Universal.
