# Supplementary material for: An innovative medical waste management system in a smart city using XAI and vehicle routing optimization
Source: F1000Res. 2023 Nov 21;12:1060. Originally published 2023 Aug 31. [Version 2] doi: 10.12688/f1000research.138867.2 (PMC10624954; doi:10.12688/f1000research.138867.2)
Supplement: Peer review history

# Reviewer report: Reviewer response for version 1

**DOI:** 10.5256/f1000research.152098.r218159

**Published:** 2023-11-03

**Authors:** Mustapha Ahlaqqach

**Affiliations:** 1 École Supérieure des Industries du Textile et de l'Habillement, Casablanca, Morocco

This article looks at a global approach to managing the end-of-life of medical and pharmaceutical products. This approach integrates waste collection procedures such as the use of colored bags, sensors to collect data relating to the waste collected and, behind this, XAI to process the data and contribute to the decision-making process relating to waste logistics up to the point of incineration. The authors have also used metaheuristics and simulation to optimize routes, reduce transport costs, control the risks associated with medical waste, and choose the best configuration for the waste treatment network. The manuscript is well written and well organized, with all parts of the research fully developed. The results of the new proposal for the treatment of medical and pharmaceutical waste can have a positive impact on all the stakeholders in this project (waste generators, population, public authorities and logistics operators). In this respect, the manuscript can be accepted as is for indexing in the journal F1000Research after a few minor modifications:

 Is the problem complex enough to apply the GA algorithm?

 Figure 11: add node 10 to the title

 Page 23: It would be interesting to put a review of the literature relating to TDVRPTW for MWM in TDVRPTW with two sub-models for the MWM paragraph.

 Equations (7), (8), (26) and (27) add for all k in K

 Equations (15), (16), (17), (34), (35) and (36) added for all p in P

 Page 29 (11) ensure that all available waste is collected

 Page 29 (17) also avoid sub-routes

 Page 29, fifth paragraph, line 1: comparison of algorithms (add GA and ALNS)

 Page 37: You can delete paragraphs 6 and 7 (Redandance)

 Page 38: Summarise the simulation results in a table

Is the work clearly and accurately presented and does it cite the current literature?

Yes

If applicable, is the statistical analysis and its interpretation appropriate?

Yes

Are all the source data underlying the results available to ensure full reproducibility?

Yes

Is the study design appropriate and is the work technically sound?

Yes

Are the conclusions drawn adequately supported by the results?

Yes

Are sufficient details of methods and analysis provided to allow replication by others?

Yes

Reviewer Expertise:

Logistics, Modelisation, operation research, Simulation

I confirm that I have read this submission and believe that I have an appropriate level of expertise to confirm that it is of an acceptable scientific standard.

# Response

**Published:** 2023-11-12

**Authors:** Zineb Boudanga

We appreciate your careful evaluation of our manuscript, and we're pleased to inform you that we have addressed each of your insightful comments and suggestions in the revised version of our paper. Here is a summary of how we've addressed your concerns:

 1. **Complexity of the GA Algorithm**: We have provided a more detailed explanation in the paper about why the GA algorithm is suitable for the complexity of the problem under consideration.

 2. **Figure 11**: We've updated Figure 11 to include "node 10" in the title, as per your suggestion.

 3. **Review of Literature on TDVRPTW for MWM**: In response to your comment, we have added a comprehensive review of the literature related to TDVRPTW for MWM in the relevant section on page 23.

 4. **Equations (7), (8), (26), and (27)**: We have added these equations for all k in K, as you recommended.

 5. **Equations (15), (16), (17), (34), (35), and (36):** In accordance with your suggestion, we have added these equations for all p in P.

 6. **Page 29 (11)**: We have correct the sentences of equation (11)

 7. **Page 29 (17)**: We have correct the sentences of equation (17)

 8. **Page 29, fifth paragraph**: We have added GA and ALNS to explain which comparaison.

 9. **Page 37**: We have removed paragraphs 6 and 7 to eliminate redundancy, as you recommended.

 10. **Page 38**: The simulation results are already summarized in Table 7.

 We believe these revisions have significantly improved the quality and completeness of our paper. We thank you for your valuable feedback and hope that the updated version aligns with your expectations.

# Reviewer report: Reviewer response for version 1

**DOI:** 10.5256/f1000research.152098.r212607

**Published:** 2023-10-09

**Authors:** Grigorios Kyriakopoulos

**Affiliations:** 1 School of Electrical and Computer Engineering, National Technical University of Athens, Athens, Greece

The manuscript is smoothly written and well organized having all research parts fully developed. An integrated methodology of Medical Waste Management (MWW) contained the employment of a variety of sensor-based systems, algorithms and advanced technologies, such as Global Positioning System (GPS)-enabled vehicles, and the explainable artificial intelligence (XAI) technology, towards a comprehensive understanding of the optimization of waste sorting, waste collection routes and storage, as well as the concurring treatment operations. The scheduled MWM and the subsequent creative argumentation and discussion on the research outcomes sustain novel findings and competitive advantages in terms of efficiency, safety, and environmental sustainability of the smart MWM system proposed, having also promising design applicability to similar waste management plans/methods and relevant spatial characteristics worldwide. In this respect the manuscript can be accepted for indexing at the F1000Research journal as is.

Is the work clearly and accurately presented and does it cite the current literature?

Yes

If applicable, is the statistical analysis and its interpretation appropriate?

Yes

Are all the source data underlying the results available to ensure full reproducibility?

Yes

Is the study design appropriate and is the work technically sound?

Yes

Are the conclusions drawn adequately supported by the results?

Yes

Are sufficient details of methods and analysis provided to allow replication by others?

Yes

Reviewer Expertise:

Chemical engineering, environmental engineering, renewable energy sources, analytical techniques, physico-chemical processes for environmental remediation of aqueous polluted systems, development economics, business and management, circular economy, behavioural ecology

I confirm that I have read this submission and believe that I have an appropriate level of expertise to confirm that it is of an acceptable scientific standard.
